# Effectiveness of the Hayling and Brixton Tests for Detecting Dementia, Progressive Cognitive Decline, and Mild Cognitive Impairment in Middle to Older Aged Adults: A Systematic Review and Meta-analysis

**DOI:** 10.1007/s11065-025-09658-6

**Published:** 2025-04-17

**Authors:** M. O. Palombo, A. M. Foran

**Affiliations:** School of Psychology, University of Adelaide, Adelaide, Australia

**Keywords:** Hayling and Brixton tests, Neurodegenerative disorders, Dementia, Frontotemporal, Parkinson’s, Executive function

## Abstract

The aging population is increasing the prevalence of dementia, neurodegenerative disorders, and mild cognitive impairment, which are associated with cognitive declines in executive functioning. In people with these disorders, accurate tests can aid in the early detection of executive functioning decline and facilitate access to interventions. The Hayling and Brixton tests (HBTs) are popular executive functioning tests that assess inhibitory control. The HBTs may be especially effective for detecting people with disorders that are associated with disinhibition, such as behavioral-variant frontotemporal dementia (bvFTD). However, the effectiveness of the HBTs for detecting cognitive decline in dementia, neurodegenerative disorders, and mild cognitive impairment has yet to be collated. A comprehensive search of five databases identified 50 studies that compared the HBTs performances of adults aged 40 years and over with a dementia, neurodegenerative disorder, or mild cognitive impairment (e.g., Parkinson’s disease, Alzheimer’s dementia, bvFTD) and cognitively-healthy controls. Hedges’ *g* effect sizes compared groups on the five HBTs scores (Inhibition Errors, Inhibition Reaction Time (RT), Automatic RT, Inhibition minus Automatic RT, and Brixton Errors). The disorders (combined) showed negative effects on all HBTs scores (*g* − 0.37 to − 1.13), with dementia (combined) performing the worst (*g* − 0.54 to − 1.56). Automatic RT and Inhibition Errors were the most effective scores for detecting cognitive decline in dementia (*g* − 1.55; *g* − 1.34). The dementia types performed similar after outliers were removed and only studies with low risk-of-bias were analyzed. Overall, the HBTs are effective for detecting cognitive decline in middle to older aged adults, especially those with dementia. However, no score type can be recommended for differentiating the dementia types, such as AD and bvFTD.

**Supplementary Information:**

The online version contains supplementary material available at 10.1007/s11065-025-09658-6.

The prevalence of older adults is expected to more than double within the next three decades, with there being 2.1 billion people over the age of 60 years by 2050 (World Health Organization (WHO), 2020). The aging of our population will lead to an increase in age-associated diseases, such as neurodegenerative disorders (Hou et al., 2019; Rose, 2009; WHO, 2021). Dementia is the most prevalent neurodegenerative disorder, and the 60 million people currently affected will increase to nearly 140 million by 2050 (Cao et al., 2020; WHO, 2023). Memory deficits are considered to be the most common symptom of dementia; however, between 64% and 96% of affected individuals also experience declines in executive functioning (D'Onofrio et al., 2018; Sengupta et al., 2019; Swanberg et al., 2004). There are a number of higher-order cognitive abilities classified under executive functioning, such as inhibitory control (which is referred to as disinhibition when impaired), task initiation, self-monitoring, and goal-directed behaviors (Krueger et al., 2011).

Declines in executive functioning are typically associated with frontal lobe damage but can also arise when there is widespread damage to the cerebral cortex (Diamond, 2013; Friedman & Robbins, 2022). In Alzheimer’s dementia (AD), diffuse cortical damage and frontal degeneration contribute to declines in executive functioning, while damage to the mesial temporal lobes is primarily responsible for the characteristic memory impairments (Guarino et al., 2019; Jones & Graff-Radford, 2021; McKhann et al., 2011; Stopford et al., 2012). Declines in executive functioning are also attributed to the accumulation of alpha-synuclein protein known as Lewy bodies in Lewy body dementia (LBD; Haikal et al., 2024). Of the dementias, frontotemporal dementia (FTD) has the most marked frontal degeneration, which results in language and behavioral changes (Bang et al., 2015; Rascovsky et al., 2011). Behavioral-variant FTD (bvFTD) is the most common FTD subtype and it is associated with damage to the medial orbitofrontal regions, which results in impaired executive functioning (Goodman et al., 2017; Hornberger et al., 2010). One of the executive functions that is particularly vulnerable to decline in bvFTD is inhibition (Magrath Guimet et al., 2021). Consequently, the *Diagnostic and Statistical Manual of Mental Disorders, 5th edition* (DSM-5) includes behavioral disinhibition in the diagnostic criteria for major or mild frontotemporal neurocognitive disorder (American Psychiatric Association, 2013). Additionally, declines in social behavior, personality, empathy, and insight are also common in bvFTD (Barker et al., 2022; Dodich et al., 2018; Rascovsky et al., 2011). The marked frontal lobe damage has led to executive functioning tests, especially those measuring inhibition, being used in clinical assessments to assist in the detection of bvFTD and to differentiate it from other dementia subtypes (Chen et al., 2020; Leslie et al., 2016; Musa et al., 2020; Ramanan et al., 2017; Roca et al., 2013; Salmon & Stuss, 2013; Schubert et al., 2016). Semantic dementia (SD) is a less common FTD subtype, which is characterized by language impairment, as well as declines in executive fucntioning (Neary et al., 2000; Seelaar et al., 2011).

People with other neurodegenerative disorders, such as Parkinson’s disease (PD), motor neuron disease (MND), multiple sclerosis (MS), and Huntington’s disease (HD), can also experience dementia or milder cognitive impairments, with declines in executive functioning particularly common (Benedict et al., 2020; Crockford et al., 2018; Hanagasi et al., 2017; McColgan & Tabrizi, 2018; Migliaccio et al., 2020; Pender et al., 2020; Robbins & Cools, 2014). Infections that cause progressive neurological changes, including human immunodeficiency virus (HIV) and prion disease, are also associated with declines in executive functioning (Brew & Chan, 2014; Caine et al., 2015; Deeks et al., 2015). Additionally, declines in executive functioning are observed in individuals with cognitive deficits who do not meet the diagnostic criteria for dementia, and are classified as having mild cognitive impairment (MCI; Chehrehnegar et al., 2020; Petersen, 2004). For the purpose of this meta-analysis, the above disorders will be collectively referred to as the “disorder group” because they are the most common causes of cognitive decline in middle to older aged adults (Benedict et al., 2020; Brew & Chan, 2014; Caine et al., 2015; Crockford et al., 2018; Hanagasi et al., 2017; Lamptey et al., 2022; Liang et al., 2021; McColgan & Tabrizi, 2018; Migliaccio et al., 2020; National Institute for Health and Care Excellence (NICE), 2020; Peplow et al., 2022; Robbins & Cools, 2014; Westervelt, 2015; WHO, 2023; Yegla et al., 2019).

The detection of executive functioning impairments in people with dementia and other disorders that are associated with cognitive decline may be particularly useful for identifying those who need support and supervision in activities of daily living (Munakata et al., 2011). Specifically, individuals with disinhibition may benefit from interventions that can reduce the likelihood of them engaging in harmful actions such as wandering, disruptive vocalizations, impulsive retorts, and inappropriate sexual behaviors (Cipriani et al., 2020). In addition, given disinhibition is common in bvFTD, its detection may assist in differentiating this dementia type from those dementias that have more pronounced memory impairments, such as AD (Borson & Chodosh, 2014; Mariano et al., 2020). The diagnosis of a specific dementia type can then lead to more tailored interventions, education, management, and recommendations for future planning, which can improve the overall quality of care and outcomes for affected individuals (Borson & Chodosh, 2014; Loi et al., 2023; Mariano et al., 2020).

There are numerous executive functioning tests that assess inhibitory control such as the Trail Making Test, Verbal Fluency Test, Stroop Test, and Wisconsin Card Sorting Test (Strauss et al., 2006). Among these, the Hayling and Brixton tests (HBTs) have been gaining clinical popularity since their publication in 1997 (Cervera-Crespo and González-Alvarez, 2017; Mole et al., 2020). The HBTs comprise two tests, the Hayling Test (referred to as the Hayling Sentence Completion Test) and the Brixton Test (referred to as the Brixton Spatial Awareness Test), which measure inhibitory control, verbal initiation, and cognitive flexibility (Burgess & Shallice, 1997). Unlike many other tests, the HBTs measure both verbal and non-verbal aspects of executive functioning (the Hayling Test and Brixton test, respectively; Bielak et al., 2006). Additionally, bedside administration and the ability to complete the Brixton Test with non-verbal responses (pointing) make the HBTs suitable for a wide range of people (Rao et al., 2021; Wall et al., 2017). The HBTs are typically sold together (Goldstein & McNeil, 2004), although the Hayling Test alone is being increasingly employed in assessments for diagnosing dementia, as well as in dementia research (Chen et al., 2020; Díaz-Rivera et al., 2023; deSouza et al., 2022; Martyr et al., 2019; Matias-Guiu et al., 2019; Vestberg et al., 2019; Wong et al., 2019).

The Hayling Test instructs participants to complete 30 sentences as fast as possible with the first 15 sentences requiring the insertion of a related word (Part A, e.g., *the job was easy most of the* [insert related word: *time*]) and the second set of 15 sentences requiring the insertion of an unrelated word (Part B, e.g., *the captain wanted to stay with the sinking* [insert unrelated word: *sky*]). Performance on Part A measures the time to produce a response (reaction time; RT) and is referred to as Automatic RT. Part B measures the time to inhibit an automatic response and provide an unrelated response, and is referred to as Inhibition RT. The number of incorrect responses or failures to provide an unrelated word is also recorded, which is referred to as Inhibition Errors. A difference score is derived by subtracting the Automatic RT score from Inhibition RT score, denoted as B-A RT, which measures the time for the participant to provide an unrelated word instead of completing a sentence in a sensible manner (i.e., efficiency of inhibiting). The Brixton Test involves showing participants a series of spatial patterns (55 in total) and asking them to anticipate or guess the subsequent item in the sequence, which assesses rule detection and attainment, as well as set shifting and inhibitory control (Burgess and Shallice, 1997; Spitoni et al., 2018).

The effectiveness of HBTs in differentiating bvFTD from other dementias is debatable (Hornberger et al., 2010; Mariano et al., 2020; O’Callaghan et al., 2013; Ramanan et al., 2017; Santillo et al., 2016; Schubert et al., 2016). However, modified versions of the Hayling Test are included in the FRONTIER and INECO executive screening batteries and are used to track bvFTD progression and distinguish it from other dementia types (Konstantinopoulou et al., 2024; Leslie et al., 2016; Torralva et al., 2009). Likewise, there are mixed findings regarding the effectiveness of the HBTs for detecting the cognitive decline that is associated with PD, MND, HD, MS, HIV, prion disease, AD, SD, and LBD (Athanasiou, 2020; Belleville et al., 2006; Ghosh et al., 2013; Johns et al., 2009; Hornberger et al., 2010; Larsen et al., 2015; Mariano et al., 2020; Martyr et al., 2019; O’Callaghan et al., 2013; Santillo et al., 2016). There is now a body of research comparing the HBTs performances of adults 40 years of age and over who are experiencing progressive cognitive decline and their cognitively healthy peers; however, the collective findings have yet to be evaluated. Consequently, our understanding of whether the HBTs are an effective test for detecting cognitive decline and whether a specific score is best for detecting certain dementia types (e.g., Inhibition Errors in bvFTD) remains limited. The current meta-analysis examined the effectiveness of the HBTs for detecting the cognitive decline that is associated with dementia (AD, bvFTD, LBD, SD), neurodegenerative and other progressive disorders (PD, MND, HD, MS, HIV, prion disease), and MCI in middle to older aged adults. The dementias were a particular focus due to their clinical significance, and Inhibition Errors performances were of special interest given disinhibition is characteristic of bvFTD.

## Methods

This meta-analysis followed the Preferred Reporting Items for Systematic Reviews and Meta-Analyses (PRISMA) guidelines (see Supplementary Table S1 for PRISMA checklist; Page et al., 2021a, 2021b).

### Search Strategy and Eligibility Criteria

The study protocol was registered with the International Prospective Register of Systematic Reviews (PROSPERO CRD42023407342). The PsycINFO, EMBASE, MEDLINE, PubMed, and Scopus databases were searched for research published from 1990 to 23 January 2024 that compared the HBTs performance in middle to older aged adults to cognitively healthy controls. This approach ensured that all relevant studies were captured since the HBTs were published in 1997. A specialist research librarian was consulted in formulating the search terms for each database (see Supplementary Table S2). The selection of search terms was based on what had been observed in the existing literature and utilized a combination of general and specific terms to ensure both common and less commonly used terminology for the HBTs were captured. Additionally, citation searching was conducted by examining the reference lists of relevant studies and conference proceedings, as well as through the Scopus and Google Scholar databases.

The studies had to meet the following criteria to be included in this meta-analysis: (1) report a Hayling and/or Brixton Test score; (2) include samples of adults (mean age + 1 *SD* > 39 years), one of which was diagnosed with a disorder that is associated with progressive cognitive decline (i.e., PD, MND, HD, MS, HIV, prion disease, AD, bvFTD, LBD, SD, or MCI; see Supplementary Table S3 for accepted diagnostic criteria) and the other comprising a cognitively healthy control group; (3) contain data enabling the computation of Hedges’ *g* effect sizes (e.g., means and *SD*s, Cohen’s *d*, *t*-test, *F*-test, exact *p*-value, and partial eta squared); and (4) be published in a peer-reviewed journal in either English or Italian language. The first author screened all studies for eligibility, with the second author additionally reviewing studies where eligibility was questionable. An independent research assistant reviewed 10% of the title and abstracts, which suggested there was high inter-rater agreement (*k* = 80; *α* = 0.98).

The following Hayling Test scores were recorded. Automatic RT: the time (reaction time, RT; seconds) required for the participant to say a word that is semantically related to the sentence, which completes it in a sensible way, with a max. of 60 seconds permitted per sentence for a total of 15 sentences. Inhibition RT: the time (seconds) required for the participant to find a semantically unrelated word, which does not complete the sentence in a sensible way, with a max. of 60 seconds permitted per sentence for a total of 15 sentences. Inhibition Errors: the number of related or somewhat related words that were provided when semantically unrelated words were required, with a max. of 45 errors possible. B-A score: the reaction time difference (seconds) between Inhibition RT and Automatic RT. The Brixton Test score was recorded in the form of errors (namely, Brixton Errors), which measures the number of incorrect responses made in predicting the next item in a pattern, with a maximum of 55 errors possible. Higher scores on any of the HBTs conditions indicate a worse performance.

Studies were excluded from the meta-analysis if (1) the HBTs were used to diagnose the disorder, this was enforced in an attempt to avoid criterion contamination, which could artificially inflate differences between the disorder group(s) and healthy controls; (2) it was a case-study; (3) the authors were unable to provide the required data, unable to be located, or failed to respond to emails; and (4) samples were overlapping or the independence of the data could not be assured. Corresponding authors were contacted if studies reported standardized HBTs scores (i.e., scaled scores) or had missing or ambiguous data, or there were concerns regarding overlapping samples. Where authors failed to respond, or could not be contacted, a conservative approach was taken whereby studies were assumed to not be independent and only the latest samples in the most recent publication were included in this meta-analysis.

### Data Extraction and Coding

The following data was extracted from each study using a standardized template (see Supplementary Table S4): (1) study details (author(s), year, country); (2) type of disorder that is associated with cognitive decline including the diagnostic criteria used and disease duration; (3) type of control (i.e., healthy matched controls, family members, caregivers) and how they were determined to be cognitively healthy (e.g., interview, cognitive screens); (4) recruitment source for both the disorder group(s) and controls (community, primary care, specialist clinic, not specified); (5) participant selection (random, consecutive, convenient, retrospective, not specified); (6) study selection criteria (e.g., exclusion of participants with head injuries or psychiatric illness); (7) demographic details (age, sex, education); (8) cognitive screening scores (i.e., Addenbrooke Cognitive Examination (ACE), Mini Mental State Examination (MMSE), Montreal Cognitive Assessment (MoCA)); and (9) Hayling and/or Brixton test scores for the disorders and cognitively healthy control groups.

Baseline or pretreatment scores were recorded if participants were assessed on multiple occasions. Means and standard deviations were estimated for demographic data and test scores from medians and/or ranges using the method recommended by Hozo et al. (2005). Studies that provided subgroup data which were not applicable to this meta-analysis (e.g., Parkinson’s disease-mild cognitive impairment and Parkinson’s disease-healthy cognition) were combined utilizing Hozo et al.’s (2005) method. A small number of studies reported the individual Hayling Test Category A (related words) or B (somewhat related) for Inhibition Errors, and for the purpose of this meta-analysis these two scores were combined as per the manual’s instructions (Category A errors multiplied by three + Category B errors; Burgess & Shallice, 1997).

### Study Risk-of-Bias

Meta-analyses assess the included studies for risk-of-bias because poorly conducted studies are more likely to produce low-quality or biased data (Spencer and Brassey, 2017). To accomplish this, the Foran and colleagues’ Risk-of-Bias Tool (2021; See Supplementary Figure S1) was applied to all included studies. This tool was chosen because it was adapted from the Quality Assessment of Diagnostic Accuracy Studies-2 (QUADAS-2; Whiting et al., 2011) specifically for evaluating the presence of bias in studies using cognitive measures for people with neurodegenerative disorders. This risk-of-bias tool allows blinding to be excluded when selecting low risk-of-bias studies, which is necessary given many disorders that are associated with cognitive decline “unblind” themselves due to their overt symptoms and the HBTs were not often the primary focus of the research. A low risk-of-bias study was determined based on three domains: (1) Sampling: the disorder group(s) had to be recruited randomly or consecutively with recruitment sources and demographic variables specified and the effect of the age and education of the controls investigated (or the controls were matched to the disorder group); (2) Diagnostic verification: published reference criteria were used for diagnosing people with a dementia, the disorder associated with progressive cognitive decline, or MCI (e.g., Rascovsky et al. (2011) for bvFTD; see Supplementary Table S3 for accepted diagnostic criteria) and additionally the controls were objectively evaluated to be cognitively healthy utilizing a cognitive screen (i.e., MMSE); and (3) Attrition: sample size remained the same, or the authors provided an explanation for any reduction in the number of participants when reporting the Hayling and/or Brixton test scores. Studies were classified into three categories: (1) low risk-of-bias if they met all four criteria; (2) moderate risk-of-bias if they partially met one of the four criteria (i.e., age was matched but not education, not all participants were recruited randomly or consecutively); and (3) high/unknown risk-of-bias if they did not meet two or more criteria or failed to provide the diagnostic reference criteria. The Risk-of-bias VISualization tool (robvis; McGuiness & Higgins, 2021) was used to summarize the risk-of-bias and generate traffic light and summary plots (see Supplementary Figure S2 and Fig. 2).

Study risk-of-bias was additionally examined in a subgroup analysis, which compared low and moderate-to-high/unknown risk-of-bias studies, with the pooled effect sizes for the low risk-of-bias studies considered to be significantly different if they fell outside the 95% confidence interval of the effect sizes for the moderate-to-high/unknown risk-of-bias studies. The effects for score types and disorder types that were of particular interest (e.g., Inhibition Errors in AD and bvFTD) were also computed using only low risk-of-bias studies, data permitting.

### Data Analysis

IBM SPSS Statistics (Version 27, 2020) was used to analyze sample and study characteristics, including age, sex, country, recruitment source, and diagnostic criteria. Effect sizes were computed using the Comprehensive Meta-Analysis software (CMA Version 4; Borenstein et al., 2022) and forest plots were generated using Microsoft Excel. Hedges’ *g* effect sizes were calculated using a random-effects model to investigate differences in HBTs scores between the disorder and cognitively healthy control groups along with 95% confidence intervals and prediction intervals (s). Confidence intervals provide information about the precision of the estimated effect size and prediction intervals provide information about both the effect and the dispersion of effects from the included studies, neither of which were adjusted for in this study (Borenstein, 2023). Prediction intervals are recommended where there are 10 or more studies and therefore were computed if data permitted (Borenstein, 2023). A negative *g* indicated poorer performance for the disorder group(s) because higher scores on the Automatic RT, Inhibition RT, Inhibition Errors, B-A RT, and Brixton Errors indicate a worse performance. Effect sizes of *g* = 0.2, 0.5, 0.8, 2.0, and 4.0 equate to small, medium, large, very large, and extremely large effects, respectively (Hopkins et al., 2009). A *p* value of < 0.05 was used to assess statistical significance. Between-study heterogeneity in the effect size was investigated using *Q*, which measures the distribution of observed effects; *Tau*^2^ (τ), which estimates the variance in true effects; and *I*^2^, which represents the ratio of true effects to error variance (Borenstein et al., 2017, 2021).

Effect sizes, prediction intervals, and heterogeneity statistics were computed for each score. First, the complete dataset was used to provide an estimate of the overall effectiveness of the HBTs scores for detecting middle to older aged people with a dementia, another disorder that is associated with progressive cognitive decline or mild cognitive impairment (disorder group). This combined disorder group was analyzed because it may indicate the utility of the HBTs for detecting declines in executive functioning in patients presenting to general medical settings. MCI was included in this combined disorder group because most people with MCI are eventually diagnosed with a neurodegenerative disorder, in particular dementia (Chen et al., 2018). For example, although the annual conversion rate from MCI to dementia ranges from 10 to 19%, approximately 70% of individuals progress to dementia within 6 years (Espinosa et al., 2013; Platero et al., 2021; Tábuas-Pereira et al., 2016). Second, the dataset was used to provide an estimate of the effectiveness of the HBTs scores for detecting cognitive decline in people with dementia (combined; AD, bvFTD, LBD, and SD). If present, outlier studies were removed and analyses were re-run to investigate their impact on the observed effect sizes and heterogeneity. Subgroup analyses comparing the disorders and dementia types were not possible because a single cognitively healthy control group was typically used in studies that investigated more than one disorder type (the assumption of independence was not met). However, effect sizes were provided for each disorder in a forest plot (including PD, MND, HD, MS, HIV, prion disease, AD, bvFTD, LBD, SD, and MCI) to allow for a visual comparison. The effects for the disorder groups were considered significantly different if the point estimate of one group fell outside the 95% CIs for the other (Bowden & Finch, 2017). AD and bvFTD were presented consecutively in the forest plot to investigate if larger effects were seen for the bvFTD group.

Subgroup analyses were conducted to investigate the influence of study risk-of-bias, with the included studies categorized as “low” or “moderate-to-high/unknown” when all disorders (combined) and only dementia (combined) were analyzed. Additionally, effects were computed using only low risk-of-bias studies to determine if the pattern of effects remained similar, data permitting. Additionally, if data permitted, meta-regressions investigated whether patients’ age, sex, education, and disease duration and severity influenced the observed effect sizes for the HBTs scores.

Publication bias was assessed using the Duval and Tweedie (2000)’s trim-and-fill method. Missing studies on the right side of the funnel plot indicated contrary findings (Rothstein et al., 2005). Publication bias was considered absent if no studies were trimmed or there was a trivial difference between observed and adjusted effect sizes; this provided an estimate of the overall effect size(s) that might have resulted if studies with potentially suppressed or unpublished results were included (Borenstein, 2019).

## Results

### Study and Sample Characteristics

**Fig. 1:**
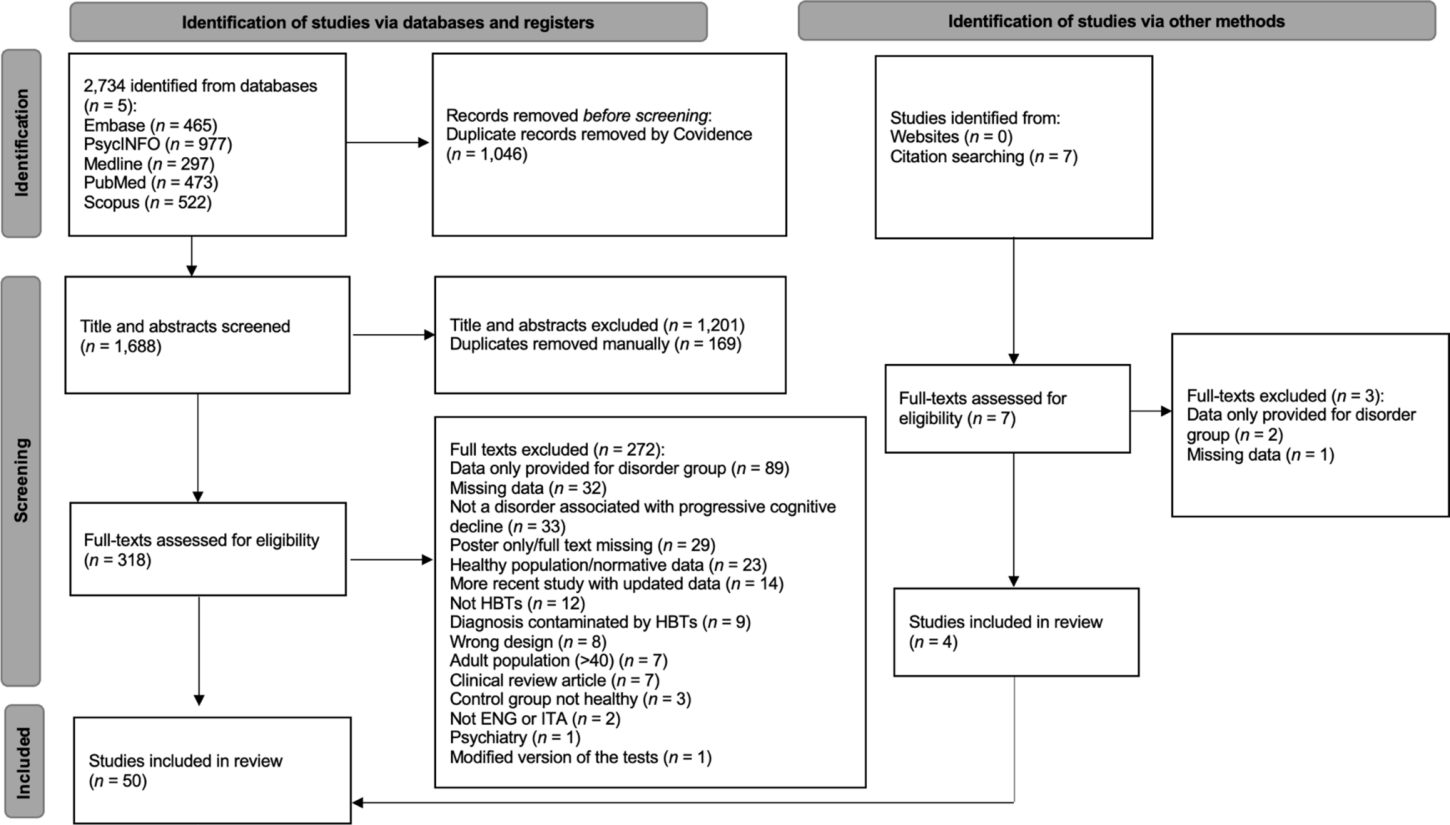
PRISMA flowchart of screening process

The literature search yielded 2734 studies, of which 1215 were duplicates. At the initial title and abstract screening, 1201 records were excluded (see Fig. 1 for PRISMA chart), leaving 318 studies in the full-text review, of which 272 studies did not meet the inclusion criteria. Additional supplementary searches conducted via Google Scholar, Scopus, and reference searching identified four eligible studies. Thirty-five authors were contacted for data or study clarification and of these 17 did not respond (Supplementary Table S7).

The final sample comprised 50 studies (see Supplementary Table S5 for individual study details): 1739 participants with dementia, a disorder associated with progressive cognitive decline or MCI (disorder group); and 2166 cognitively healthy controls (see Table 1). Overall, the disorder group had significantly higher average age (*M* = 66 years) and a higher proportion of males (55%) compared to the cognitively healthy control group (*M* = 65 years, 43% males). Additionally, the disorder group had a lower level of education (*M* = 12 years) compared to the controls (*M* = 13 years). Cognitive screening scores were significantly lower in the disorder group compared to controls, with the average MMSE score for the disorder group above the recommended cutoff cognitive impairment, but the average ACE score was in the impaired range (cutoff values: MMSE < 24; ACE < 81; Jubb & Evans, 2015; Mitchell, 2009). Most of the participants in the disorder group were recruited through specialist or outpatient clinics and controls were sourced from the community. The Hayling Test (*N*_*studies*_ = 32) was the most commonly reported, followed by the Brixton Test (*N*_*studies*_ = 13) with both tests (HBTs; *N*_*studies*_ = 5) being reported by fewer studies. Inhibition Errors was the most commonly reported score (*N*_*studies*_ = 39), followed by Inhibition RT (*N*_*studies*_ = 29), Automatic RT (*N*_*studies*_ = 21), Brixton Errors (*N*_*studies*_ = 19), and B-A RT (*N*_*studies*_ = 13). The most common diagnoses were PD (*N*_*studies*_ = 16) followed by AD (*N*_*studies*_ = 12), bvFTD (*N*_*studies*_ = 10), MND (*N*_*studies*_ = 9), MCI (*N*_*studies*_ = 7), SD (*N*_*studies*_ = 4), HD (*N*_*studies*_ = 3), LBD (*N*_*studies*_ = 1), HIV (*N*_*studies*_ = 1), MS (*N*_*studies*_ = 1), and prion disease (*N*_*studies*_ = 1).

**Table 1:** Summary sample demographic characteristics for the included studies

	Disorder group			Controls				
	*N*_*studies*_	*N*_*participants*_	*M(SD)*	*N*_*studies*_	*N*_*controls*_	*M(SD)*	*t/χ*^2^	*p*
Total sample	50	1739		50	2166			
age	50	1739	66.0 (7.7)	50	2166	64.8 (7.6)	4.9	0.00
sex (M/F)	50	962/777		49	930/1185		49.2	0.00
education (yrs)	39	1440	11.8 (2.3)	38	1715	12.7 (2.6)	10.2	0.00
disease duration (yrs)	29	822	5.1 (2.9)					
MMSE	26	908	25.8 (2.2)	18	652	28.9 (0.4)	35.6	0.00
ACE	10	353	74.9 (8.5)	10	409	92.7 (5.2)	35.4	0.00
MoCA	5	119	22.6 (4.1)	5	130	26.6 (1.2)	10.6	0.00
Recruitment source								
community	1	50		26	1383			
primary care	0	0						
specialist clinic	35	1299						
inpatient	5	167						
mixed	1	20		6	205			
family				4	137			
unstated	7	182		14	428			
Hayling Test								
Automatic RT	21	441		21	715			
Inhibition RT	29	639		29	879			
Inhibition Errors	39	800		39	1124			
B-A RT	13	328		13	364			
Brixton Test								
Errors	19	597		19	766			
Parkinsonian disorders								
age	16	409	66.8 (4.1)	16	480	65.7 (3.4)	4.4	0.01
sex (M/F)	16	247/163		16	225/255		15.9	0.00
education (yrs)	14	379	12.1 (2.9)	13	323	12.9 (2.9)	3.6	0.00
disease duration (yrs)	14		6.5 (3.2)					
MMSE	8	218	27.8 (0.9)	6	167	29.0 (0.4)	16.1	0.00
ACE	4	110	76.1 (11.8)	4	148	95.0 (1.5)	19.3	0.00
MoCA	4	87	24.6 (2.0)	4	70	27.0 (1.4)	8.5	0.00
Motor neuron disease								
age	9	288	59.7 (2.4)	9	242	60.2 (2.6)	2.3	0.04
sex (M/F)	9	172/115		9	124/117		3.8	0.05
education (yrs)	6	190	13.3 (0.7)	6	160	14.1 (0.6)	11.5	0.00
disease duration (yrs)	7	205	2.5 (0.5)					
MMSE	2	41	27.5 (0.7)	2	40	29.3 (0.2)	15.8	0.00
ACE	3	88	79.3 (2.7)	3	72	85.9 (7.9)	6.8	0.00
HIV-1								
age	1	30	46.7 (10.7)	1	30	48.5 (9.9)		
sex (M/F)	1	25/5		1	30	24/6		
education (yrs)	1	30	6.0 (6.0)	1	30	5.0 (3.0)		
Huntington's disease								
age	3	106	51.1 (0.5)	3	89	45.3 (4.6)	11.8	0.00
sex (M/F)	3	60/46		3	41/48		2.2	0.14
education (yrs)	2	66	12.4 (0.8)	2	57	12.6 (2.1)	0.7	0.99
disease duration (yrs)	1	18	4.5 (2.9)					
MMSE	2	66	26.7 (1.8)	1	39	29.3 (0.8)	10.2	0.00
Prion disease								
age	1	40	51.4 (11.4)	1	33	48. 9(13.4)		
sex (M/F)	1	24/16		1	17/16			
Multiple sclerosis								
age	1	42	42.7 (10.3)	1	21	40.5 (10.7)		
sex (M/F)	1	8/34		1	4/17			
disease duration (yrs)	1	42	5.1 (3.2)					
Mild cognitive impairment								
age	7	280	71.0 (3.6)	7	420	69.6 (2.5)	5.7	0.00
sex (M/F)	7	143/137		7	179/241		4.8	0.03
education (yrs)	7	280	10.7 (2.9)	7	420	10.8 (3.7)	0.4	0.67
MMSE	4	155	27.2 (1.2)	3	77	28.7 (0.3)	14.7	0.00
Alzheimer’s dementia								
age	12	251	74.1 (4.4)	12	313	70.8 (4.1)	9.2	0.00
sex (M/F)	12	111/140		12	117/196		2.7	0.10
education (yrs)	11	224	11.0 (1.4)	11	287	12.5 (1.7)	10.7	0.00
MMSE	9	220	23.3 (1.7)	8	205	28.7 (0.3)	44.8	0.00
ACE	3	60	65.7 (4.8)	3	96	93.6 (2.4)	48.2	0.00
Behavioral-variant frontotemporal dementia								
age	10	217	66.8 (2.8)	10	310	67.2 (3.4)	1.4	0.15
sex (M/F)	10	138/79		9	104/166		30.3	0.00
education (yrs)	9	200	12.2 (2.1)	9	234	13.4 (1.2)	7.4	0.00
disease duration (yrs)	4	73	5.7 (2.4)					
MMSE	8	181	25.0 (1.0)	5	124	29.1 (0.3)	44.3	0.00
ACE	2	71	76.6 (3.7)	2	62	95.3 (1.4)		
Semantic dementia								
age	4	38	67.0 (2.6)	4	175	67.6 (2.4)	1.4	0.17
sex (M/F)	4	13/25		4	69/106		0.4	0.55
education (yrs)	3	33	12.1 (1.0)	3	99	14.6 (1.2)	10.8	0.00
disease duration (yrs)	2	30	5.2 (2.2)					
MMSE	2	12	27.3 (1.8)					
ACE	1	24	81.4 (10.1)	1	31	95.8 (3.1)		
Lewy body dementia								
age	1	15	73.3 (5.7)	1	20	71.4 (5.0)		
sex (M/F)	1	7/8		1	11/9			
education (yrs)	1	15	10.1 (3.8)	1	20	12.5 (2.0)		
MMSE	1	15	23.8 (4.6)	1	20			

*N*_*studies*_*=* number of studies; *N*_*participants*_*=* number of participants; *M(SD) =* weighted mean (standard deviation); *N*_*controls*_*=* number of controls; *t/χ*^*2*^*= t*-test or Chi-square statistic; *p =* probability value; *M/F =* male/female; *yrs =* years; *MMSE =* Mini Mental Status Examination; *ACE=* Addenbrooke’s Cognitive Examination; *MoCA =* Montreal cognitive assessment; *RT =* reaction time; *HIV-1 =* human immunodeficiency virus

### Risk-of-Bias in Individual Studies

**Fig. 2:**
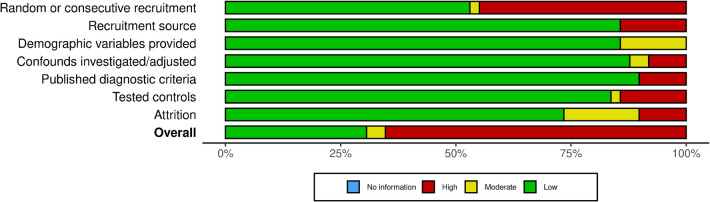
Summary risk-of-bias plot illustrating the proportion of individual studies meeting the Readapted QUADAS-2 Criteria (Foran et al., 2021)

A summary of the study risk-of-bias analysis using the adapted QUADAS-2 criteria is presented in Fig. 2 (Foran et al., 2021; Whiting et al., 2011), with 30% of the included studies classified as low risk-of-bias, 4% as moderate, and 66% as high-or-unknown risk-of-bias. Supplementary Table S6 provides the study risk-of-bias classifications for individual studies. A strength of many included studies (*N*_*studies*_ = 44) was the use of published reference criteria to diagnose the disorders and cognitive screening measures for inclusion as a healthy control. Most studies were classified as moderate or high-or-unknown risk-of-bias due to the lack of random or consecutive recruitment, with the majority using convenient sampling.

### Hayling and Brixton Tests Performance: Disorders (Combined)

**Fig. 3:**
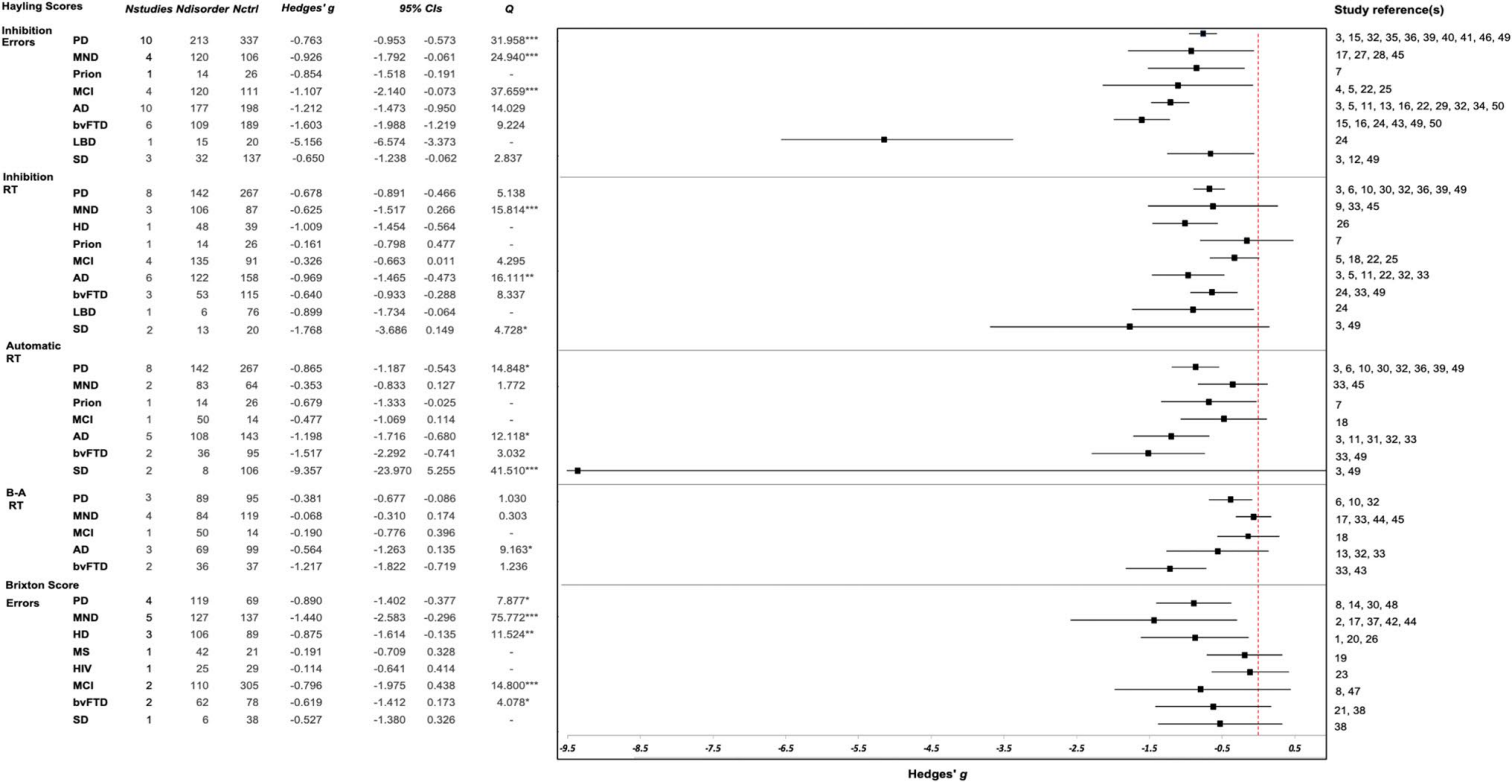
Forest plot showing Hedges’ *g* effect sizes for the disorder group, grouped by disease classification and score type. *Hayling* = Hayling Sentence Completion test; *Brixton* = Brixton Test; *N*_*studies*_= number of studies; *N*_*disorder*_= number of participants with a disorder; *N*_*ctrl*_= number of cognitively healthy controls; *95% CIs *= 95% confidence intervals; *Q *= distribution of observed effects; *PD* = Parkinsonian disorders; *MND =* motor neuron disorders; *HD* = Huntington’s disease; *MS* = multiple sclerosis; *HIV =* human immunodeficiency virus; *MCI =* mild cognitive impairment; *AD =* Alzheimer’s dementia; *bvFTD =* Behavioral-variant frontotemporal dementia; *SD =* semantic dementia; *LBD =* Lewy body dementia; *RT =* reaction time. *p value < 0.05, **p value < 0.01, ***p value < 0.001

Table 2 presents a summary of the mean Hedges’ *g* effect sizes for the five HBTs scores (Inhibition Errors, Inhibition RT, Automatic RT, B-A RT, and Brixton Errors) for all disorders (combined). The analysis revealed large effect sizes for Inhibition Errors (*g* = − 1.129), Automatic RT (*g* = − 0.888), and Brixton Errors (*g* = − 0.831), a medium effect size for Inhibition RT (*g* = − 0.705), and a small effect size for the B-A RT (*g* = − 0.366) score. All effect sizes were statistically significant (*p* < 0.05) and consistently in the negative direction, indicating the disorder group scored lower on the HBTs compared to the cognitively healthy controls. Wide prediction intervals were observed for all scores, which indicated variation in effects across studies. In addition, the inclusion of zero in the prediction intervals indicated several people in the disorder group performed similar to their cognitively healthy peers, which is in keeping with the heterogeneous nature of this grouping and that not all individuals with these disorders are cognitively impaired. Significant heterogeneity was observed for all five scores (see Table 2 for *Q* statistics). It was noted that the heterogeneity in the effect sizes for the Inhibition (Errors and RT) and Automatic RT scores could be influenced by outlier scores from two studies that were used to compute effect sizes for LBD and SD (Fig. 3 illustrates the extremely large effect sizes for LBD and SD). However, after removing these studies from the overall analysis, significant heterogeneity remained.

**Table 2:** Hayling and Brixton tests: Hedges’* g* effect sizes and heterogeneity statistics for the disorders (combined) and dementia (combined)

Test	Score type	N_studies_	g	95% prediction intervals		Q	I^2^	Tau	Tau^2^
Disorder group									
Hayling	Inhibition Errors	28	− 1.129	− 2.401	0.142	162.42***	83.38	0.61	0.37
Hayling	Inhibition RT	19	− 0.705	− 1.556	0.146	61.43***	70.70	0.39	0.15
Hayling	Automatic RT	14	− 0.888	− 2.077	0.300	65.01***	80.00	0.52	0.27
Hayling	B-A RT	10	− 0.366	− 0.976	0.244	17.78*	49.39	0.24	0.06
Brixton	Errors	17	− 0.831	− 2.241	0.579	119.77***	86.64	0.64	0.41
Dementia									
Hayling	Inhibition Errors	15	− 1.341	− 2.485	− 0.197	52.33***	73.25	0.51	0.26
Hayling	Inhibition RT	8	− 0.956	− 2.123	0.211	23.44***	70.14	0.44	0.19
Hayling	Automatic RT	6	− 1.559	− 3.676	0.558	33.38***	85.02	0.69	0.48
Hayling	B-A RT	4	− 0.714	− 3.721	2.293	17.88***	83.22	0.61	0.38
Brixton	Errors	2	− 0.537			3.12	67.99	0.35	0.12

*N*_*studies*_*=* number of studies; *g =* Hedges’ *g*; *95% PIs =* 95% prediction intervals; *Q =* distribution of observed effects; *I*^*2*^*=* ratio of true effect to error variance; *Tau*^*2*^*=* variance in the true effects; *RT =* reaction time

*p value < 0.05, **p value < 0.01, ***p value < 0.001

### Hayling and Brixton Tests Performance: Dementia

Table 2 summarizes the mean effect sizes for all dementias combined, all of which were in the expected direction (negative) and statistically significant (*p* < 0.05), indicating the dementia group consistently had poorer HBTs performances compared to cognitively healthy controls. Unsurprisingly, the dementia types, characterized by their hallmark cognitive difficulties, exhibited larger effect sizes on the Hayling Test than the disorders that have more pronounced physical symptoms. Among the five score types, Inhibition Errors and Automatic RT were both the most commonly reported and distinguishing (*g* = − 1.341 and *g* = − 1.559, respectively), followed by Inhibition RT (*g* = − 0.956), which also had a large effect size, then B-A RT (*g* = − 0.714) and Brixton Errors (*g* = − 0.537) which had medium effect sizes. Significant heterogeneity was observed in all scores, except for Brixton Errors.

On Inhibition Errors, bvFTD (*g* = − 1.603) scored worse than the other dementia types, except for LBD, which was an outlier study. The LBD and SD outlier studies were included in the subgroup analysis; however, it is worth noting that their exclusion from the analysis for all dementia (combined) resulted in the heterogeneity no longer being statistically significant for Inhibition Errors, which suggests the dementia types performed similarly (*N*_*studies*_ = 13, *g* = − 1.187, 95% CI − 1.395 to − 0.979, *Q* = 19.861, *p* = 0.07). In addition, the Inhibition Errors point estimate for AD overlapped the confidence intervals for bvFTD very slightly, which suggests the difference between these dementia types was not significant (i.e., Hedges’ *g* point estimate for the bvFTD group did not fall outside the 95% CIs for the AD group).

On Inhibition RT, SD had the worst performance (*g* = − 1.768), followed by AD (*g* = − 0.969); however, all the dementia types had point estimates that overlapped with other dementia types CIs, which suggests they performed similarly. On Automatic RT, significant heterogeneity remained, even after the outlier SD study was removed, with bvFTD performing the lowest (*g* = − 1.517) followed by AD (*g* = − 1.198); however, the point estimates and CIs again overlapped suggesting the difference between these dementia types was not significant. On B-A RT, the point estimates and CIs for bvFTD (*g* = − 1.217) and AD (*g* = − 0.564) also overlapped. On Brixton Errors, heterogeneity was not statistically significant and although the two dementia types appeared to perform similarly (*g* = − 0.619 and *g* = − 0.527, respectively), it is worthwhile noting there were only two bvFTD studies and one SD study. The heterogeneity that remained unaccounted for in the Hayling Test scores for many dementia types prompted subgroup analyses examining study risk-of-bias as a potential cause of variation.

### Risk-of-Bias and Meta Regressions

**Fig. 4:**
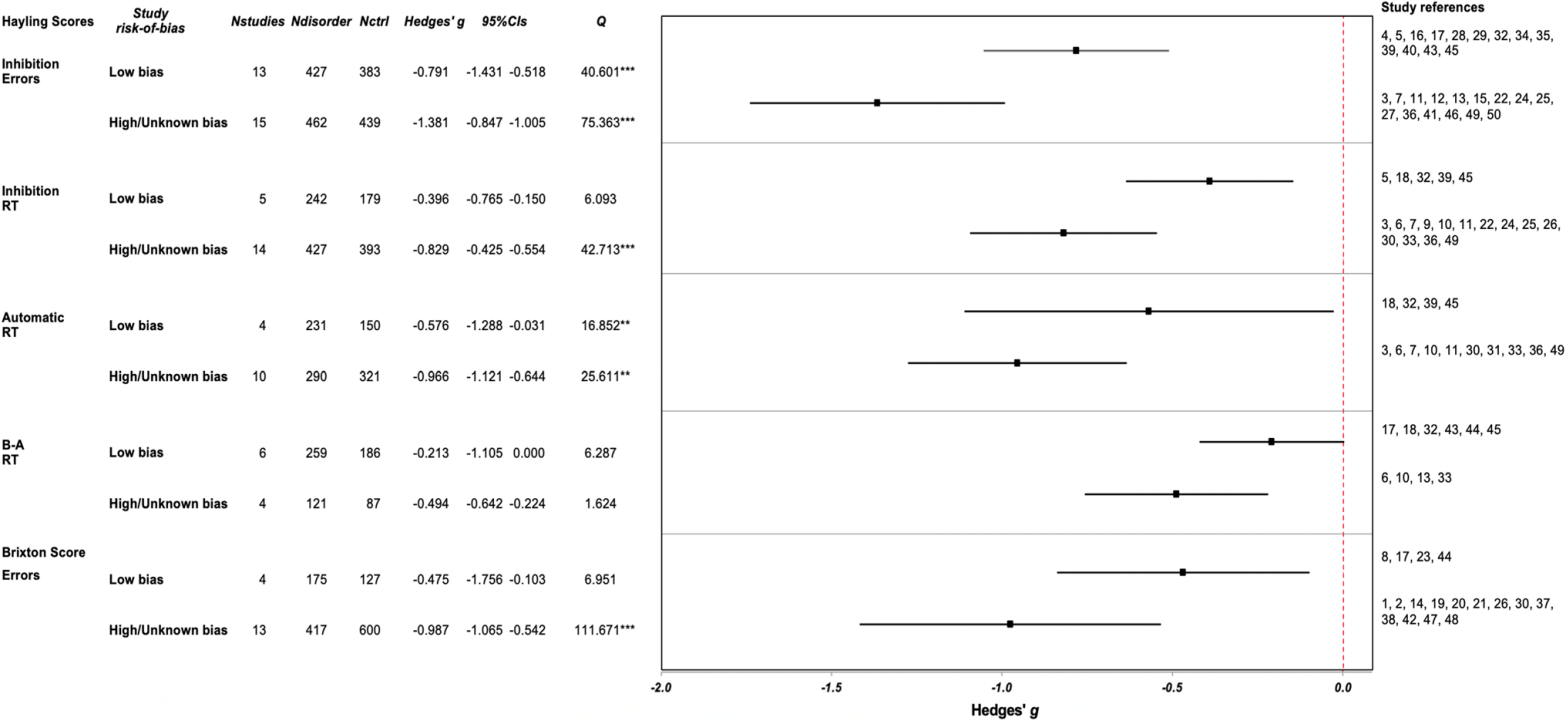
Forest plot showing Hedges’ *g* effect sizes for all disorders (combined) on the HBTs scores, grouped by study risk-of-bias. *Hayling =* Hayling Test; *N*_*studies*_= number of studies; *N*_*disorder*_= number of participants with disorders; *N*_*ctrl*_= number of cognitively healthy controls; *95% CIs *= 95% confidence intervals; *Q *= distribution of observed effects. *p value < 0.05, **p value < 0.01, ***p value < 0.001

A subgroup analysis investigated study risk-of-bias for all disorders (combined). Grouping the 15 studies that were classified as “low” and the 35 studies that were categorized as “moderate-to-high/unknown” risk-of-bias accounted for a significant amount of the heterogeneity in Inhibition Errors and Automatic RT but not for the Inhibition RT, B-A RT, and Brixton Errors scores. Figure 4 shows that the low risk-of-bias studies had smaller effect sizes than the “moderate-to-high/unknown” risk-of-bias studies, especially for the Inhibition Errors (*N*_*studies*_ = 13) and Brixton Errors (*N*_*studies*_ = 4), followed by Inhibition RT (*N*_*studies*_ = 5), Automatic RT (*N*_*studies*_ = 4), and B-A RT (*N*_*studies*_ = 6) scores; however, the effects remained medium to large.

The risk-of-bias subgroup analysis was also conducted using only the dementia types (combined) and it accounted for a significant amount of the variability in Inhibition Errors, Inhibition RT, and B-A RT. The effect sizes for studies with low risk-of-bias were smaller in magnitude, for example Automatic RT (*N*_*studies*_ = 1, *g* = − 1.308), Inhibition Errors (*N*_*studies*_ = 6, *g* = − 1.117), Inhibition RT (*N*_*studies*_ = 2, *g* = − 0.690), and B-A RT (*N*_*studies*_ = 2, *g* = − 0.557), than those for studies with moderate-to-high/unknown risk-of-bias. However, the point estimates and CIs for the low and moderate-to-high/unknown studies overlapped for all of the Hayling Test scores and significant heterogeneity remained in the low risk-of-bias studies for Inhibition Errors and the Automatic RT score. When only low risk-of-bias studies were analyzed, the effects for Inhibition Errors were similar for AD (*N*_*studies*_ = 5, *g* = − 1.154, 95% CI = − 1.533 to − 0.766) and bvFTD (*N*_*studies*_ = 2, *g* = − 1.263, 95% CI = − 1.776 to − 0.750), with effects for LBD (*N*_*studies*_ = 0) and SD (*N*_*studies*_ = 0) not able to be computed due to an insufficient number of studies.

A meta-regression was conducted for Inhibition Errors in PD and AD because there were sufficient study numbers (*N*_*studies*_ > 9). Age, sex, education, and disease duration did not influence the PD group performance on Inhibition Errors; age (*N*_*studies*_ = 9, *R*^2^ = 0.00, *p* = 0.79); sex (*N*_*studies*_ = 9, *R*^2^ = 0.00, *p* = 0.32); patient education (*N*_*studies*_ = 9, *R*^2^ = 0.00, *p* = 0.55); and disease duration (*N*_*studies*_ = 9, *R*^2^ = 0.03, *p* = 0.27). Age, sex, and education also did not influence the AD group performance on Inhibition Errors; age (*N*_*studies*_ = 9, *R*^2^ = 0.08, *p* = 0.31); sex (*N*_*studies*_ = 9, *R*^2^ = 0.00, *p* = 0.38), and patient education (*N*_*studies*_ = 9, *R*^2^ = 0.00, *p* = 0.55). Unfortunately, this meta-analysis was not able to investigate sources of variation in the other disorders or HBTs scores due to insufficient study numbers. Additionally, other inherent differences in the patient cohorts, such as disease duration and severity, parts of the brain affected, and severity of atrophy, could not be investigated because most studies did not report these variables in a uniform manner.

### Publication Bias

PD and AD were the most studied disorders and Inhibition Errors was the most commonly reported HBTs score. Consequently, publication bias was investigated using the Duval and Tweedie (2000) trim-and-fill method for Inhibition Errors in PD and AD (see Supplementary Figure S3). The funnel plot was asymmetrical and there was one missing study inserted for PD and three missing studies inserted for AD, which suggests publication bias may have inflated the effect size for Inhibition Errors in these disorders.

## Discussion

This meta-analysis pooled data from 50 studies to assess the effectiveness of the HBTs for detecting cognitive decline in middle to older aged adults. Dementia was of particular interest due to its clinical significance and because the HBTs are used to differentiate dementia types, especially bvFTD from AD. PD, AD, bvFTD, MND, and MCI were the most commonly reported disorders, and the Hayling Test was the most frequently administered. The HBTs scores were effective at discriminating the combined disorders group from the cognitively healthy controls, and in particular the effect sizes for Inhibition Errors remained large when only low risk-of-bias studies were analyzed. This result supports the use of Inhibition Errors for detecting the cognitive decline that may be associated with a variety of progressive disorders in middle to older aged adults. The effectiveness of the HBTs for detecting disorders that are associated with progressive cognitive decline in middle to older aged adults appears to rival other popular executive functioning tests such as the Stroop Test and Trail Making Test (Coemans et al., 2022; Prado et al., 2019); however, Foran et al. (2021) found larger effect sizes for sorting tests. According to the Cattell-Horn-Carroll Theory, executive functioning encompasses a broad range of cognitive processes that include both visual and verbal components, and consequently clinicians often administer multiple tests to assess it comprehensively (Floyd et al., 2010; Jewsbury et al., 2017). The HBTs may therefore be used to complement other executive functioning tests, for example the Hayling Test may be administered alongside the Stroop to assess verbal inhibition.

In regard to dementia, large effects were seen on the HBTs, with the effects for Automatic RT and Inhibition Errors remaining large when only low risk-of-bias studies were analyzed. Therefore, the effectiveness of Automatic RT and Inhibition Errors for detecting the cognitive decline that is associated with dementia in middle to older aged adults was supported by this meta-analysis. The tendency for people with dementia to be slower to respond on the Hayling Test (as indicated by the Automatic RT score) may be attributed to a multitude of factors that are common in many dementias, such as slowed cognitive processing, word finding difficulties, and comprehension difficulties (Raimo et al., 2024). Although Inhibition Errors and Automatic RT were very effective at detecting dementias generally, the differences between people with AD and bvFTD were not especially marked and were not significant when outlier studies were removed and only low risk-of-bias studies were analyzed. This finding was unexpected given behavioral disinhibition is a diagnostic feature of bvFTD (American Psychiatric Association, 2013). Performances by people with AD and bvFTD were also similar on sorting tests, which likewise measure inhibition (for meta-analysis, see Foran et al., 2021). Collectively, these findings are in keeping with the clinical overlap observed between AD and bvFTD (Blennerhassett et al., 2014; Musa et al., 2020). The findings are also consistent with the view that that standalone cognitive measures are inadequate for differentiating between AD and bvFTD (Bruun et al., 2018; Overbeek et al., 2020; Piguet et al., 2017).

Our interpretation of the most effective HBTs score is limited by the available data, and not all scores and disorders were reported equally (e.g., no Brixton Errors scores were reported for AD). A key methodological limitation relates to only 10% of studies being inter-rated at the title and abstract screening stage, and the PRISMA guidelines recommend at least two independent reviewers are employed to reduce the risk of missing relevant studies (Page et al., 2021a, 2021b). Another limitation relates to the poor response rate from authors, which resulted in potentially relevant studies being excluded because the data was unavailable, or there were concerns regarding overlapping datasets and diagnostic/criterion contamination. Additionally, a multivariate meta-analysis would have more accurately detected differences between the dementia groups (available in R Statistical Software; v4.4.1; R Core Team, 2024) than the approach of comparing point estimates and 95% confidence intervals that was used in this study (Bowden & Finch, 2017). The effect sizes in this meta-analysis may have been overestimated due to the disorder group being older and less educated than the cognitively healthy controls, given these demographic factors are known to correlate with lower cognitive performance (Lövdén et al., 2020). However, the meta-regressions showed that age and education did not affect Inhibition Errors in the PD and AD groups. Similarly, variables such as disease duration and severity, which can affect cognitive performance in progressive diseases, were not able to be analyzed due to inconsistent reporting (Small, 2016). Also, publication bias was detected for Inhibition Errors in PD and AD, which suggests there may be unpublished studies that could reduce the observed effect sizes.

Future research should focus on investigating HBTs scores in people with the types of dementia that are often differentiated using cognitive assessments, such as AD and bvFTD, and cannot be detected using other methods (e.g., vascular changes on neuroimaging for VaD). Studies should be large enough to detect small to medium effects, composed of people who meet the diagnostic criteria for AD and bvFTD, and who are early in the disease course. Additionally, the dataset in this study could be updated with multiple independent reviewers involved in the screening, risk-of-bias evaluation, and data-entry stages to improve the confidence in these findings. A multivariate meta-analysis could also be conducted with the updated dataset to determine if the observed heterogeneity in the HBTs scores can be attributed to the type of dementia, namely AD and bvFTD.

In conclusion, Inhibition Errors from the Hayling Test is particularly effective for detecting the cognitive decline that is associated with a variety of progressive disorders in middle to older aged adults. In dementia specifically, both Automatic RT and Inhibition Errors from the Hayling Test are especially effective for detecting the cognitive decline. However, people with different types of dementias perform similar on the HBTs, which suggests that there is no score type that can be recommended for differentiating the dementia types, such as AD and bvFTD.

## Supplementary Information

Below is the link to the electronic supplementary material.

Supplementary file1 (DOCX 1.51 MB)

## Data Availability

The datasets developed and used during the current study are available from the corresponding author upon reasonable request.
