# Supplementary material for: Effectiveness of the Hayling and Brixton Tests for Detecting Dementia, Progressive Cognitive Decline, and Mild Cognitive Impairment in Middle to Older Aged Adults: A Systematic Review and Meta-analysis
Source: Neuropsychol Rev. 2025 Apr 17;36(1):43–60. doi: 10.1007/s11065-025-09658-6 (PMC13279680; doi:10.1007/s11065-025-09658-6)

**Supplementary File**

**Effectiveness of the Hayling and Brixton Tests for detecting dementia, progressive cognitive decline and mild cognitive impairment in middle to older aged adults: A systematic review and meta-analysis**

Neuropsychology Review

M O Palombo (https://orcid.org/0009-0002-8220-1203)

A M Foran (https://orcid.org/0000-0002-8515-9197)

School of Psychology

University of Adelaide

Corresponding author email: [amie.foran@adelaide.edu.au](mailto:amie.foran@adelaide.edu.au)

Supplementary Table S1. PRISMA Checklist

| **Section and Topic** | **Item #** | **Checklist item** | **Location where item is reported** |
| --- | --- | --- | --- |
| **TITLE** | | |  |
| Title | 1 | Identify the report as a systematic review. | 1 |
| **ABSTRACT** | | |  |
| Abstract | 2 | See the PRISMA 2020 for Abstracts checklist. | 2 |
| **INTRODUCTION** | | |  |
| Rationale | 3 | Describe the rationale for the review in the context of existing knowledge. | 5-7 |
| Objectives | 4 | Provide an explicit statement of the objective(s) or question(s) the review addresses. | 7 |
| **METHODS** | | |  |
| Eligibility criteria | 5 | Specify the inclusion and exclusion criteria for the review and how studies were grouped for the syntheses. | 8-9 |
| Information sources | 6 | Specify all databases, registers, websites, organisations, reference lists and other sources searched or consulted to identify studies. Specify the date when each source was last searched or consulted. | 7-8 |
| Search strategy | 7 | Present the full search strategies for all databases, registers and websites, including any filters and limits used. | Supplementary Table S2  5 |
| Selection process | 8 | Specify the methods used to decide whether a study met the inclusion criteria of the review, including how many reviewers screened each record and each report retrieved, whether they worked independently, and if applicable, details of automation tools used in the process. | 8-9 |
| Data collection process | 9 | Specify the methods used to collect data from reports, including how many reviewers collected data from each report, whether they worked independently, any processes for obtaining or confirming data from study investigators, and if applicable, details of automation tools used in the process. | 8-10 |
| Data items | 10a | List and define all outcomes for which data were sought. Specify whether all results that were compatible with each outcome domain in each study were sought (e.g. for all measures, time points, analyses), and if not, the methods used to decide which results to collect. | 9 |
|  | 10b | List and define all other variables for which data were sought (e.g. participant and intervention characteristics, funding sources). Describe any assumptions made about any missing or unclear information. | 9-10 |
| Study risk of bias assessment | 11 | Specify the methods used to assess risk of bias in the included studies, including details of the tool(s) used, how many reviewers assessed each study and whether they worked independently, and if applicable, details of automation tools used in the process. | 10-11 |
| Effect measures | 12 | Specify for each outcome the effect measure(s) (e.g. risk ratio, mean difference) used in the synthesis or presentation of results. | 11 |
| Synthesis methods | 13a | Describe the processes used to decide which studies were eligible for each synthesis (e.g. tabulating the study intervention characteristics and comparing against the planned groups for each synthesis (item #5)). | 9-10 |
|  | 13b | Describe any methods required to prepare the data for presentation or synthesis, such as handling of missing summary statistics, or data conversions. | 10-11 |
|  | 13c | Describe any methods used to tabulate or visually display results of individual studies and syntheses. | 11 |
|  | 13d | Describe any methods used to synthesize results and provide a rationale for the choice(s). If meta-analysis was performed, describe the model(s), method(s) to identify the presence and extent of statistical heterogeneity, and software package(s) used. | 11-13 |
|  | 13e | Describe any methods used to explore possible causes of heterogeneity among study results (e.g. subgroup analysis, meta-regression). | 13 |
|  | 13f | Describe any sensitivity analyses conducted to assess robustness of the synthesized results. | N/A |
| Reporting bias assessment | 14 | Describe any methods used to assess risk of bias due to missing results in a synthesis (arising from reporting biases). | 13 |
| Certainty assessment | 15 | Describe any methods used to assess certainty (or confidence) in the body of evidence for an outcome. | 12 |
| **RESULTS** | | |  |
| Study selection | 16a | Describe the results of the search and selection process, from the number of records identified in the search to the number of studies included in the review, ideally using a flow diagram. | 13-15  Figure 1 |
|  | 16b | Cite studies that might appear to meet the inclusion criteria, but which were excluded, and explain why they were excluded. | Figure 1  15 |
| Study characteristics | 17 | Cite each included study and present its characteristics. | Supplementary Table S5  11 |
| Risk of bias in studies | 18 | Present assessments of risk of bias for each included study. | Supplementary Table S6 and Figure S2  16-20 |
| Results of individual studies | 19 | For all outcomes, present, for each study: (a) summary statistics for each group (where appropriate) and (b) an effect estimate and its precision (e.g. confidence/credible interval), ideally using structured tables or plots. | Table 2  20 |
| Results of syntheses | 20a | For each synthesis, briefly summarise the characteristics and risk of bias among contributing studies. | 19 |
|  | 20b | Present results of all statistical syntheses conducted. If meta-analysis was done, present for each the summary estimate and its precision (e.g. confidence/credible interval) and measures of statistical heterogeneity. If comparing groups, describe the direction of the effect. | Figure 3  23 |
|  | 20c | Present results of all investigations of possible causes of heterogeneity among study results. | Table 2  20 |
|  | 20d | Present results of all sensitivity analyses conducted to assess the robustness of the synthesized results. | N/A |
| Reporting biases | 21 | Present assessments of risk of bias due to missing results (arising from reporting biases) for each synthesis assessed. | Figure 2  19 |
| Certainty of evidence | 22 | Present assessments of certainty (or confidence) in the body of evidence for each outcome assessed. | Figure 4  25 |
| **DISCUSSION** | | |  |
| Discussion | 23a | Provide a general interpretation of the results in the context of other evidence. | 27-28 |
|  | 23b | Discuss any limitations of the evidence included in the review. | 28-29 |
|  | 23c | Discuss any limitations of the review processes used. | 29 |
|  | 23d | Discuss implications of the results for practice, policy, and future research. | 28-30 |
| **OTHER INFORMATION** | | |  |
| Registration and protocol | 24a | Provide registration information for the review, including register name and registration number, or state that the review was not registered. | 7 |
|  | 24b | Indicate where the review protocol can be accessed, or state that a protocol was not prepared. | 7 |
|  | 24c | Describe and explain any amendments to information provided at registration or in the protocol. | N/A |
| Support | 25 | Describe sources of financial or non-financial support for the review, and the role of the funders or sponsors in the review. | N/A |
| Competing interests | 26 | Declare any competing interests of review authors. | N/A |
| Availability of data, code and other materials | 27 | Report which of the following are publicly available and where they can be found: template data collection forms; data extracted from included studies; data used for all analyses; analytic code; any other materials used in the review. | Supplementary Table S4  10 |

*From:*  Page, M. J., McKenzie, J. E., Bossuyt, P. M., Boutron, I., Hoffmann, T. C., Mulrow, C. D., Shamseer, L., Tetzlaff, J. M., Akl, E. A., Brennan, S. E., Chou, R., Glanville, J., Grimshaw, J. M., Hróbjartsson, A., Lalu, M. M., Li, T., Loder, E. W., Mayo-Wilson, E., McDonald, S., McGuinness, L. A., … & Moher, D. (2021). The PRISMA 2020 statement: an updated guideline for reporting systematic reviews. *BMJ (Clinical research ed.)*, *372*, n71. <https://doi.org/10.1136/bmj.n71>

Supplementary Table S2. Logic grids

**PsycINFO & Medline**

| Hayling and Brixton tests |
| --- |
| (Hayling sentence completion* or Hayling* or Hayling* task* or hayling* test* or Brixton spatial anticipation* or Brixton* or Brixton Test* or Brixton task* or "Hayling* and Brixton*" or "Hayling* test of inhibitory control" or Spatial anticipation* or Hayling* Brixton* or "Hayling and Brixton*" or Hayling*-Brixton* or Hayling* sentence completion or Brixton Spatial Anticipation or Brixton spatial rule attainment).mp |

**Embase**

| Hayling and Brixton tests |
| --- |
| Hayling sentence completion test.mp OR Hayling sentence completion task.mp OR Hayling task.mp OR Hayling Test.mp OR Brixton spatial anticipation test.mp OR Brixton Test.mp OR Brixton task.mp OR "Hayling and Brixton Tests".mp OR Sentence completion test.mp OR Spatial anticipation test.mp OR Hayling Brixton Tests.mp OR "Hayling Test of inhibitory control".mp OR "Hayling and Brixton executive tests".mp OR Hayling-Brixton Tests.mp OR Hayling Brixton Tests.mp OR Hayling* sentence completion.mp OR Brixton Spatial Anticipation.mp OR "Brixton spatial rule attainment".mp |

**Pubmed**

| Hayling and Brixton tests |
| --- |
| Hayling sentence completion*[All fields] or Hayling*[All fields] or Hayling task*[All fields] or Haylings task*[All fields] or hayling* test*[All fields] or haylings test*[All fields] or Brixton spatial anticipation*[All fields] or Brixton*[All fields] or Brixton Test*[All fields] or Brixton task*[All fields] or "Hayling and Brixton"[All fields] or "Hayling and Brixton Test"[All fields] or "Hayling and Brixton task"[All fields] or "Haylings and Brixton"[All fields] or "Haylings and Brixton Test"[All fields] or "Haylings and Brixton task"[All fields] or "Hayling Test of inhibitory control"[All fields] or Spatial anticipation*[All fields] or Hayling Brixton*[All fields] or Haylings Brixton*[All fields] or Hayling-Brixton*[All fields] or Haylings-Brixton*[All fields] or Hayling sentence completion[All fields] or Haylings sentence completion[All fields] or Brixton Spatial Anticipation[All fields] or Brixton spatial rule attainment[All fields] |

**Scopus**

| Hayling and Brixton tests |
| --- |
| "Hayling sentence completion*" or Hayling* or "Hayling* task*" or "hayling* test*" or "Brixton spatial anticipation*" or Brixton* or "Brixton Test*" or "Brixton task*" or "Hayling* and Brixton*" or "Hayling* test of inhibitory control" or "Spatial anticipation*" or "Hayling* Brixton*" or "Hayling and Brixton*" or "Hayling*-Brixton*" or "Hayling* sentence completion" or "Brixton Spatial Anticipation" or "Brixton spatial rule attainment" |

Supplementary Figure S1. Risk-of-bias Assessment (Foran et al., 2021)

**The study clearly reported:**

| **Sampling** | that participants with neurodegenerative disorders were recruited consecutively or randomly | NO  NO  **High or unknown bias risk**  NO  NO  NO  NO  NO |
| --- | --- | --- |
|  | where participants with a neurodegenerative disorder were recruited (e.g., clinic/specialist, GP, community, inpatient, etc.) |  |
|  | the age & education for the neurodegenerative/MCI sample |  |
|  | the age & education for the controls or reported that they were matched-controls (age *&* education) |  |
|  | YES  **Low risk of sampling bias** |  |
| **Verification** | that published criteria^1^ were used to diagnose the neurodegenerative disorder, *unless* it was published prior to accepted published criteria and clear patient inclusion criteria were provided |  |
|  | controls were excluded if they were known or suspected to have a neurological or psychiatric condition  YES |  |
|  | **Low risk of verification bias** |  |
| **Attrition** | there was no reduction in the number of participants who had reported sorting test scores or, if attrition did occur, it was due to unavoidable reasons (e.g., death) |  |
|  | YES |  |
|  | **Low risk of attrition bias** |  |

Supplementary Table S3. Published Diagnostic Criteria for Each of the Neurodegenerative Disorders (adapted from Foran et al., 2021)

|  | **Neurodegenerative disorder** | **Diagnostic criteria** |
| --- | --- | --- |
| Parkinsonian | Parkinson’s Disease (PD) | Barbeau (1986); Gelb, Oliver, & Gilman (1999); Hughes, Daniel, Kilford, & Lees (1992); Lang & Lozano (1998); Litvan et al., (2012); Postuma et al., (2015); Ward & Gibb (1990) |
|  | Multiple System Atrophy (MSA) | [Quinn (1989)](#_ENREF_26) |
|  | Corticobasal Syndrome (CBS) | Armstrong et al., (2013); Mathew et al., (2012); Shelley et al., (2009) |
|  | Progressive Supranuclear Palsy (PSP) | Hoglinger et al., (2017); Litvan et al., (1996) [Shibayama et al., (2007)](#_ENREF_29) |
| Motor Neuron Disease | Motor Neuron Disease (MND) &  Amyotropic Lateral Sclerosis (LS) | Andersen et al., (2005); Beghi et al., (2002); Brooks (1994); Brooks, Miller, Swash, & Munsat (2000); World Federation of Neurology Research Group on Neuromuscular Diseases Subcommittee on Motor Neuron Disease. Airlie House guidelines. Therapeutic trials in amyotrophic lateral sclerosis. Airlie House "Therapeutic Trials in ALS" Workshop Contributors 1995 |
| MCI | Mild Cognitive Impairment (MCI) | Crook et al., (1986); Morris et al., (2006); Petersen (2004); Petersen et al., (2001); Petersen & Negash (2008); Winblad et al., (2004) |
| Other NeuroD | Normal Pressure Hydrocephalus (NPH) | Lumber puncture |
|  | Huntington's Disease (HD)  Multiple Sclerosis (MS) | CAG repeat genetic testing  Poser et al., (1983) |
|  | Human immunodeficiency virus (HIV+)  Prion disease | Seropositive  Prion protein gene sequencing |
| Dementia | Dementia of the Alzheimer’s Type (AD) | American Psychiatric Association (2013); McKhann et al., (1984); Morris et al., (1989); Morris et al., (2006); Weintraub et al., (2009) |
|  | Behavioral variant fronto-temporal degeneration/dementia (bvFTD) | Lund-Manchester (1994); Neary et al., (1998); Neary, Snowden, & Mann (2000); Neary, Snowden, Northen, & Goulding (1988); Rascovsky et al., (2007); Rascovsky et al., (2011) |
|  | Lewy Body Dementia (LBD) | McKeith (2006); McKeith et al., (2017); McKeith et al., (1996); McKeith, Perry, Fairbairn, Jabeen, & Perry (1992) |
|  | Semantic Dementia (SD) | Gorno-Tempini et al., (2011); [Neary et al., (1998)](#_ENREF_19) |
|  | Primary Progressive Aphasia (PPA) | Gorno-Tempini et al., 2011; [Neary et al., (1998)](#_ENREF_19) |
|  | Dementia general or not otherwise specified (NOS) | Diagnostic and Statistical Manual of Mental Disorders, National Institute of Neurological Disorders and Stroke |

*Note.* NeuroD = Neurodegenerative disorder, CAG = Cytosine-Adenine-Guanine.

Supplementary Table S4. Data Extraction Form


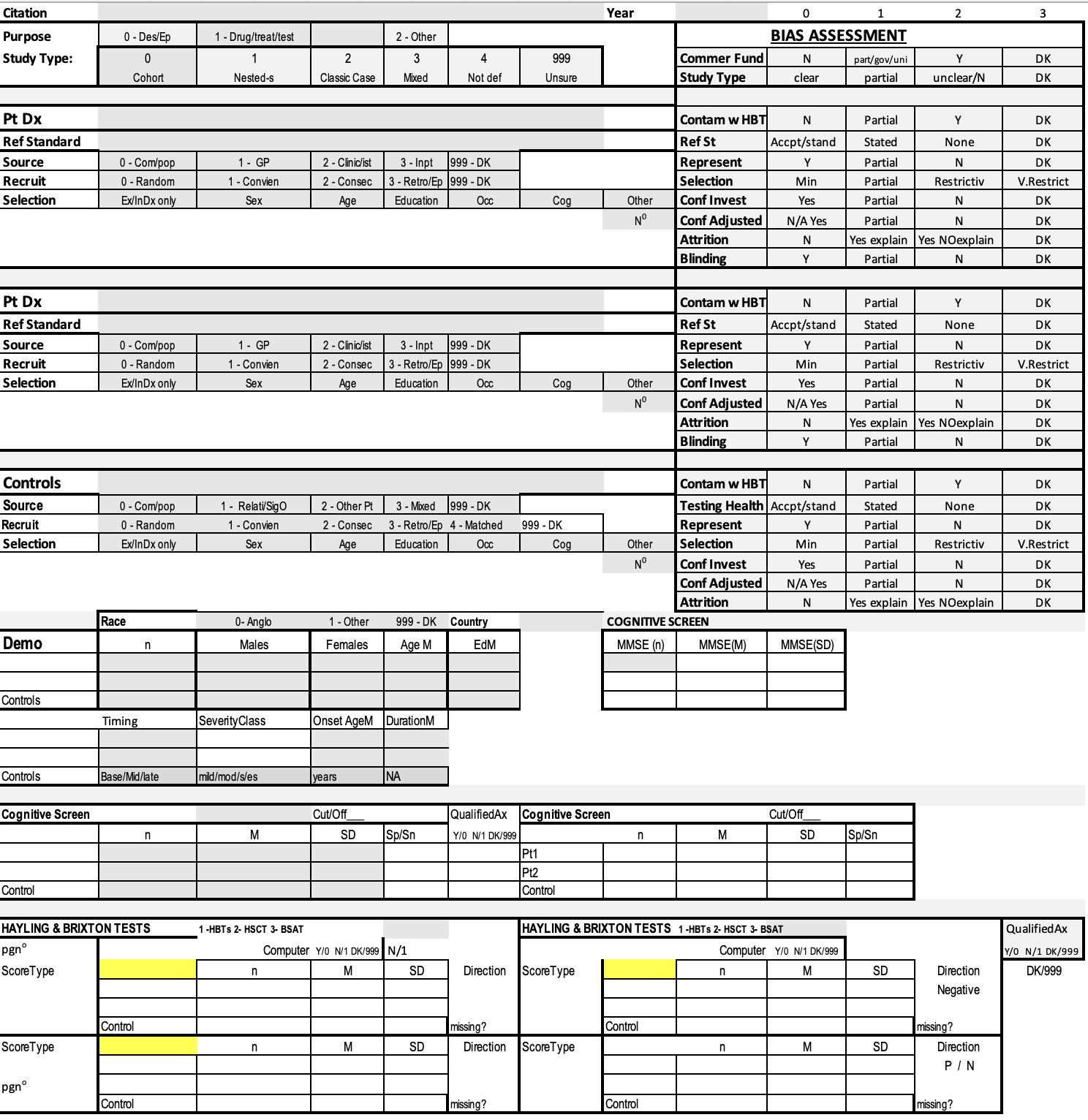


Supplementary Table S5. Summary Study Characteristics

| *reference* | *Study* | *ND* | *Published*  *diagnostic criteria* | *Test type* | *Recruitment source* | *Age*  *M(SD)* | *Educat-ion* | *Illness durati-on* | *MMSE/MoCA* | *ACE* | *Country* | *N_neuroD_* | *N_controls_* |
| --- | --- | --- | --- | --- | --- | --- | --- | --- | --- | --- | --- | --- | --- |
| 1 | Allain et al., 2011 | HD | CAG genetic testing | Brixton | Clinic/Specialist/Unit | 50.7 (8.8) | 11.8 (2.5) | 4.5 (2.9) | 25.4 (1.8) |  | France | 18 | 18 |
| 2 | Andrews et al., 2017 | ALS | Brooks, Miller, Swash & Munsat (2000) | Brixton | Clinic/Specialist/Unit | 64.0  (9.7) |  | 2.98 (2.9) |  | 79.3 (4.8) | Australia | 33 | 22 |
| 3 | Barker et al., 2018 | PSP | Litvan et al., (2003); Respondek et al., (2013) | Hayling | Inpatient | 70.0 (6.0) | 11.2 (3.2) |  |  |  | Australia | 5 | 30 |
|  |  | AD | _not provided_ | Hayling | Inpatient | 65.7 (1.2) | 11.0 (0.8) |  |  |  | Australia | 3 | 30 |
|  |  | SD | _not provided_ | Hayling | Inpatient | 70.0 (5.0) | 11.0 (1.0) |  |  |  | Australia | 2 | 30 |
| 4 | Bastin et al., 2013 | MCI | Petersen & Negash (2008) | Hayling | Clinic/Specialist/Unit | 73.9 (6.6) | 13  (3.5) |  |  |  | Belgium | 35 | 34 |
| 5 | Belleville et al., 2007 | aMCI | Petersen (2003) | Hayling | Clinic/Specialist/Unit | 64.8 (10.8) | 14.3 (4.7) |  | 28.3 (2.0) |  | Canada | 25 | 25 |
|  |  | AD | DSM-IV, McKhann et al. (1984) | Hayling | Clinic/Specialist/Unit | 73.4 (9.2) | 11  (3.8) |  | 24.7 (3.6) |  | Canada | 19 | 19 |
| 6 | Bouquet et al., 2009 | PD | Gibb & Lees (1988) | Hayling | _not provided_ | 66.1 (7.6) | 8.5  (2.2) | 10.3 (5.8) | 27.7 (1.5) |  | France | 20 | 20 |
| 7 | Caine et al., 2018 | Prion | Prion protein gene sequencing | Hayling | Clinic/Specialist/Unit | 51.4 (11.4) |  |  |  |  | United Kingdom | 40 | 33 |
| 8 | Cammisuli & Sportiello 2017 | PD-MCI | Litvan et al. (2012) | Brixton | Inpatient | 73.1 (7.7) | 6.1 (2.9) | 2.3 (3.6) |  |  | Italy | 40 | 22 |
|  |  | aMCI | Winblad et al. (2004) | Brixton | Inpatient | 66.1 (7.6) | 6.2 (3.1) | 2.6 (3.8) |  |  | Italy | 40 | 22 |
| *reference* | *Study* | *ND* | *Published*  *diagnostic criteria* | *Test type* | *Recruitment source* | *Age*  *M(SD)* | *Educat-ion* | *Illness durati-on* | *MMSE/MoCA* | *ACE* | *Country* | *N_neuroD_* | *N_controls_* |
| 9 | Carluer et al., 2015 | ALS | Brooks et al. (2000) | Hayling | _not provided_ | 59.7 (13.0) | 9.96 (2.8) | 1.6 (0.7) |  |  | France | 23 | 23 |
| 10 | Castener et al., 2007 | PD | _not provided_ | Hayling | Inpatient | 61.5 (9.2) | 13.0 (4.8) | 12.8 (4.6) |  |  | Australia | 18 | 21 |
| 11 | Cervera-Crespo et al., 2019 | AD | DSM-IV, McKhann et al. (1984) | Hayling | _not provided_ | 79.2 (4.8) | 9.9 (4.0) |  | 23.1 (1.2) |  | Spain | 31 | 16 |
| 12 | Chen et al., 2020 | SD | Gorno-Tempini et al. (2011) | Hayling | Clinic/Specialist/Unit | 66.8 (8.9) | 12.9 (3.1) | 3.7 (2.2) |  | 81.4 (10.1) | Australia | 24 | 31 |
| 13 | Collette et al., 2002 | AD | McKhann et al. (1984) + PET | Hayling | _not provided_ | 69.0 (7.4) |  |  | 19.3 (4.2) |  | Belgium | 26 | 26 |
| 14 | Danti et al., 2015 | PD | Hughes et al. (1992), UK Brain Bank + SPECT | Brixton | _not provided_ | 63.8 (8.3) | 9.8 (3.5) | 1.6 (1.0) | 27.6 (2.3) |  | Italy | 36 | 18 |
| 15 | deSouza et al., 2022 | bvFTD | Rascovsky et al. (2011) | Hayling | Inpatient | 65.8 (9.0) | 13.0 (3.3) | 3.7 (1.8) | 25.5 (2.7) |  | Brazil | 23 | 23 |
|  |  | PSP | Hoglinger et al. (2017) | Hayling | Inpatient | 67.6 (7.8) | 13.0 (3.7) | 4.1 (1.9) | 26.1 (2.4) |  | Brazil | 21 | 23 |
| 16 | Diaz-Rivera et al., 2023 | bvFTD | Rascovsky et al. (2011) | Hayling | Clinic/Specialist/Unit | 68.2 (9.4) | 15.0 (5.0) |  | 21.8 (4.5) |  | Argentina | 14 | 30 |
|  |  | AD | McKhann et al. (1984, 2011) | Hayling | Clinic/Specialist/Unit | 73.3 (4.7) | 11.9 (4.4) |  | 15.1 (6.2) |  | Argentina | 18 | 30 |
| 17 | Girardi et al., 2011 | ALS | Brooks et al. (2000) | HBTs | Clinic/Specialist/Unit | 57.4 (15.6) | 14.4 (5.4) | 3.2 (2.5) |  |  | United Kingdom | 14 | 20 |
| 18 | Goncalves et al., 2019 | MCI | Winblad et al. (2004) | Hayling | Community | 68.6 (6.2) | 11.3 (4.5) |  |  |  | Brazil | 50 | 14 |
| 19 | Hayter et al., 2016 | MS | _not provided_ | Brixton | Clinic/Specialist/Unit | 42.7 (10.3) |  | 5.1 (3.2) |  |  | United Kingdom | 42 | 21 |
| 20 | Hendel et al., 2022 | HD | CAG genetic testing | Brixton | Clinic/Specialist/Unit | 51.7 (11.9) |  |  |  |  | Denmark | 40 | 32 |
| 21 | Hornberger et al., 2009 | bvFTD | Neary et al. (1998) | HBTs | Clinic/Specialist/Unit | 65.2 (7.3) | 11.9 (2.8) |  | 27.1 (3.0) | 79.2 (15.1) | United Kingdom | 50 | 40 |
| 22 | Jacus et al., 2018 | aMCI | Petersen (2004) | Hayling | Clinic/Specialist/Unit | 75.8 (6.9) | 8.8 (2.7) |  | 26.1 (1.7) |  | France | 20 | 20 |
| *reference* | *Study* | *ND* | *Published*  *diagnostic criteria* | *Test type* | *Recruitment source* | *Age*  *M(SD)* | *Educat-ion* | *Illness durati-on* | *MMSE/MoCA* | *ACE* | *Country* | *N_neuroD_* | *N_controls_* |
|  |  | AD | McKhann et al. (1984) + MRI | Hayling | Clinic/Specialist/Unit | 79.5 (5.2) | 9.2 (2.7) |  | 24.3 (2.6) |  | France | 20 | 20 |
| 23 | Janssen et al., 2013 | HIV-1 | Seropositive | Brixton | Clinic/Specialist/Unit | 46.7 (10.7) | 6.0 (6.0) |  |  |  | Netherlands | 30 | 30 |
| 24 | Johns et al., 2009 | LBD | McKeith et al. (2004) | Hayling | Clinic/Specialist/Unit | 73.3 (5.7) | 10.1 (3.8) |  | 23.8 (4.6) |  | Canada | 15 | 20 |
|  |  | FTD | Neary et al. (2005) | Hayling | Clinic/Specialist/Unit | 66.6 (9.0) | 11.5 (4.1) |  | 24.5 (4.9) |  | Canada | 17 | 20 |
| 25 | Johns et al., 2012 | MCI | Petersen & Negash (2008); Winblad (2004) | Hayling | Clinic/Specialist/Unit | 72.4 (8.6) | 13.1 (3.1) |  | 28.1 (1.4) |  | Canada | 40 | 32 |
| 26 | Larsen et al., 2015 | HD | CAG genetic testing | HBTs | Clinic/Specialist/Unit | 51.0 (12.8) | 13.0 (2.3) |  | 28.0 (1.8) |  | Denmark | 48 | 39 |
| 27 | Lillo et al., 2011 | ALS | Brooks et al. (2000) | Hayling | Clinic/Specialist/Unit | 59.6 (8.5) | 13.5 (3.3) | 2.0 (0.9) |  | 82.0 (16.4) | Australia | 20 | 20 |
| 28 | Lillo et al., 2020 | ALS | Awaji criteria | Hayling | Clinic/Specialist/Unit | 59.6 (8.1) | 13.3 (4.5) | 2.5 (0.5) | 27.0 (1.3) |  | Chile | 22 | 21 |
| 29 | Lozachmeur et al., 2019 | AD | McKhann et al. (2011) | Hayling | Clinic/Specialist/Unit | 73.2 (7.2) | 8.8 (2.8) |  | 22.0 (1.5) |  | France | 30 | 33 |
| 30 | Macoir et al., 2013 | PD | _not provided_ | HBTs | Clinic/Specialist/Unit | 68.3 (7.1) | 12.1 (3.7) | 8.9 (4.4) | 24.7 (2.4) |  | Canada | 15 | 15 |
| 31 | Mariano et al., 2020 | AD | McKhann et al. (2011) + brain MRI + lumbar puncture | Hayling | Clinic/Specialist/Unit | 76.0 (15.0) | 11.0 (7.0) | 3.0 (1.0) | 24.3 (2.8) |  | Brazil | 25 | 24 |
|  |  | bvFTD | Rascovsky et al. (2011) + brain MRI + lumbar puncture | Hayling | Clinic/Specialist/Unit | 68.0 (19.0) | 11.0 (4.0) | 4.0 (2.5) | 25.2 (3.5) |  | Brazil | 27 | 24 |
| 32 | Martyr et al., 2019 | AD | ICD-10 | Hayling | Clinic/Specialist/Unit | 78.4 (7.4) | 13.7 (3.5) |  | 23.1 (2.9) | 65.9 (10.5) | United Kingdom | 30 | 54 |
|  |  | PD | Hughes et al. (1992) | Hayling | Clinic/Specialist/Unit | 72.2 (8.0) | 12.7 (2.5) | 5.7 (4.4) | 29.4 (1.1) | 93.8 (4.8) | United Kingdom | 33 | 54 |
| *reference* | *Study* | *ND* | *Published*  *diagnostic criteria* | *Test type* | *Recruitment source* | *Age*  *M(SD)* | *Educat-ion* | *Illness durati-on* | *MMSE/MoCA* | *ACE* | *Country* | *N_neuroD_* | *N_controls_* |
| 33 | Matias-Guiu et al., 2019 | bvFTD | Rascovsky et al. (2011) | Hayling | Clinic/Specialist/Unit | 71.1 (7.7) | 8.2 (10.7) |  | 24.0 (4.8) |  | Spain | 19 | 19 |
|  |  | AD | McKhann et al. (2011) | Hayling | Clinic/Specialist/Unit | 72.2 (8.5) | 10.7 (5.7) |  | 24.3 (4.3) |  | Spain | 19 | 19 |
|  |  | ALS | Brooks et al. (2000) | Hayling | Clinic/Specialist/Unit | 57.9 (9.7) | 12.7 (5.1) |  | 28.0 (1.6) |  | Spain | 19 | 19 |
| 34 | Nash 2007 | AD | DSM-IV, McKhann et al. (1984) | Hayling | Mixed | 79.3 (7.2) | 11.8 (2.4) |  | 24.6 (3.6) | 70.3 (10.1) | Australia | 20 | 20 |
| 35 | O'Callaghan et al., 2013 | PD | Hughes et al. (1992), UK Brain Bank | Hayling | Clinic/Specialist/Unit | 63.8 (7.7) | 13.4 (2.6) | 5.8 (4.4) | 28.0 (2.0) |  | Australia | 50 | 27 |
| 36 | Obeso et al., 2011 | PD | Hughes et al. (1992) UK Brain Bank | Hayling | Clinic/Specialist/Unit | 55.7 (6.7) |  | 9.4 (2.9) | 28.1 (1.3) |  | United Kingdom | 18 | 29 |
| 37 | Pettit et al., 2013 | ALS | Brooks et al. (2000) | Brixton | Clinic/Specialist/Unit | 58.5 (12.8) |  | 2.8 (1.7) |  |  | United Kingdom | 30 | 30 |
| 38 | Primativo et al., 2017 | bvFTD | Rascovsky et al. (2011) | Brixton | _not provided_ | 67.7 (6.3) | 14.0 (2.6) | 9.2 (6.0) | 25.1 (3.5) |  | United Kingdom | 12 | 38 |
|  |  | SD | Gorno-Tempini et al. (2011) | Brixton | _not provided_ | 63.6 (8.1) | 12.3 (2.9) | 6.8 (2.8) | 26.0 (2.0) |  | United Kingdom | 6 | 38 |
| 39 | Robinson et al., 2021 | PD | Hughes et al. (1992) | HBTs |  | 70.2 (6.0) | 13.4 (3.3) | 8.4 (5.3) | 25.4 (3.8) |  | Australia | 21 | 22 |
| 40 | Siquier & Andres 2022 | PD | Hughes et al. (1992), UK Brain Bank | Hayling | Inpatient | 67.4 (9.7) | 13.4 (4.6) | 6.9 (4.6) | 26.5 (2.5) |  | Spain | 15 | 15 |
| 41 | Southi et al., 2018 | CBS | Matthew et al. (2012) + Shelley et al. (2009) | Hayling | Clinic/Specialist/Unit | 67.4 (7.4) | 11.8 (3.5) | 3.8 (2.2) |  | 68.6 (20.0) | Australia | 28 | 28 |
| 42 | Staios et al., 2013 | ALS | Brooks et al. (2000) | Brixton | Clinic/Specialist/Unit | 63.5 (9.7) |  | 2.7 (2.8) |  | 76.6 (3.6) | Australia | 35 | 30 |
| 43 | Tanguy et al., 2022 | bvFTD | Rascovsky et al. (2011) | Hayling | Clinic/Specialist/Unit | 64.4 (8.3) | 14.3 (4.9) | 4.4 (2.3) | 23.7 (2.6) |  | France | 17 | 18 |
| *reference* | *Study* | *ND* | *Published*  *diagnostic criteria* | *Test type* | *Recruitment source* | *Age*  *M(SD)* | *Educat-ion* | *Illness durati-on* | *MMSE/MoCA* | *ACE* | *Country* | *N_neuroD_* | *N_controls_* |
| 44 | Taylor et al., 2013 | ALS | Brooks et al. (2000) | HBTs | Clinic/Specialist/Unit | 59.8 (9.1) | 13.8 (3.3) |  |  |  | United Kingdom | 51 | 35 |
| 45 | Tjokrowijoto et al., 2023 | MND | _not provided_ | Hayling | Clinic/Specialist/Unit | 59.6 (10.0) | 12.4 (2.8) | 1.7  (1.7) |  |  | Australia | 64 | 45 |
| 46 | Tse et al., 2020 | PSP | Litvan et al. (1996); Hoglinger et al. (2017) | Hayling | Clinic/Specialist/Unit | 68.2 (4.2) | 11.8 (2.7) | 3.7 (3.1) |  | 72.0 (8.6) | Australia | 16 | 33 |
|  |  | CBS | Armstrong et al. (2013) | Hayling | Clinic/Specialist/Unit | 65.6 (7.0) | 11.6 (3.1) | 4.1 (1.7) |  | 70.1 (20.8) | Australia | 33 | 33 |
| 47 | Van den Berg et al., 2009 | MCI | _not provided_ | Brixton | Clinic/Specialist/Unit | 70.8 (9.8) | 12.3 (3.6) |  | 26.3 (2.7) |  | Netherlands | 70 | 283 |
| 48 | Vandenbossche et al., 2013 | PD | _not provided_ | Brixton | _not provided_ | 66.9 (6.6) | 19.8 (3.3) |  | 28.4 (1.3) | 9.2 (3.3) | Belgium | 28 | 14 |
| 49 | Vestberg et al., 2019 | bvFTD | Rascovsky et al. (2011) | Hayling | Clinic/Specialist/Unit | 69.8 (5.5) |  |  | 25.1 (4.3) |  | Sweden | 17 | 76 |
|  |  | PSP | Litvan et al. (1996) | Hayling | Clinic/Specialist/Unit | 67.5 (6.7) |  |  | 27.3 (2.6) |  | Sweden | 12 | 76 |
|  |  | SD | Neary et al. (1998) | Hayling | Clinic/Specialist/Unit | 67.7 (6.7) |  |  | 28.5 (1.4) |  | Sweden | 6 | 76 |
| 50 | Wong et al., 2019 | AD | McKhann et al. (2011) | Hayling | Clinic/Specialist/Unit | 67.8 (9.1) | 12.3 (2.9) | 6.5 (1.4) |  |  | Australia | 10 | 22 |
|  |  | bvFTD | Rascovsky et al. (2011) | Hayling | Clinic/Specialist/Unit | 61.5 (7.1) | 11.6 (2.3) | 5.6 (1.4) |  |  | Australia | 21 | 22 |

*Note.* ND = Neurodegenerative disorder, *M(SD)*= mean (standard deviation), MMSE = Mini Mental State Examination, MoCA = Montreal Cognitive Assessment, ACE = Addenbrooke's Cognitive Examination, *N_neuroD_* = number of participants with neurodegenerative disorders*, N_controls_* = number of controls, HBTs = Hayling and Brixton Tests, CAG = Cytosine-Adenine-Guanine, HD = Huntington's disease, ALS = amyotrophic lateral sclerosis, MCI = mild cognitive impairment, aMCI = amnestic mild cognitive impairment, AD = Alzheimer's dementia, PD = Parkinson's disease, PD-MCI = Parkinson's disease-mild cognitive impairment, SD = semantic dementia, bvFTD = behavioral variant frontotemporal dementia, PSP = progressive supranuclear palsy, MS = multiple sclerosis, HIV-1 = human immunodeficiency virus-1, LBD = Lewy body dementia, FTD = frontotemporal dementia, CBS = corticobasal syndrome, MND = motor neuron disease.

Supplementary Table S6. Risk-of-bias Assessment for Each Individual Studies Included in the Meta-analysis

|  | **Sampling** | | |  | **Blinding**** | **Verification** | | **Attrition***** |
| --- | --- | --- | --- | --- | --- | --- | --- | --- |
|  | Random or consecutive recruitment | Recruitment source | Demographic variables provided | Confounds investigated/adjusted* |  | Published diagnostic criteria | Tested controls |  |
| Allain et al., 2011 | Y | Y | Y | Y | N | Y | U | N |
| Andrews et al., 2017 | N | Y | Y | Y | N | Y | N | N |
| Barker et al., 2018 | N | Y | Y | Y | N | P | N | N |
| Bastin et al., 2013 | Y | Y | Y | Y | N | Y | Y | N |
| Belleville et al., 2007 | Y | Y | Y | Y | N | Y | Y | P |
| Bouquet et al., 2009 | U | U | Y | Y | N | Y | Y | N |
| Caine et al., 2018 | N | Y | P | Y | N | Y | Y | Y |
| Cammisuli & Sportiello 2017 | Y | Y | Y | Y | N | Y | Y | N |
| Carluer et al., 2015 | U | U | Y | Y | N | Y | Y | N |
| Castener et al., 2007 | N | Y | Y | Y | N | N | Y | N |
| Cervera-Crespo et al., 2019 | U | U | Y | U | N | Y | P | N |
| Chen et al., 2020 | N | Y | Y | Y | N | Y | U | Y |
| Collette et al., 2002 | U | U | P | Y | N | Y | Y | P |
| Danti et al., 2015 | Y | U | Y | Y | N | Y | Y | N |
| deSouza et al., 2022 | N | Y | Y | Y | N | Y | Y | N |
| Diaz-Rivera et al., 2023 | U | Y | Y | Y | N | Y | Y | N |
|  | **Sampling** | | |  | **Blinding**** | **Verification** | | **Attrition***** |
|  | Random or consecutive recruitment | Recruitment source | Demographic variables provided | Participant confounds investigated* |  | Published diagnostic criteria | Tested controls |  |
| Girardi et al., 2011 | Y | Y | Y | Y | N | Y | Y | N |
| Goncalves et al., 2019 | Y | Y | Y | Y | N | Y | Y | N |
| Hayter et al., 2016 | Y | Y | P | Y | N | N | Y | N |
| Hendel et al., 2022 | Y | Y | Y | Y | N | Y | N | N |
| Hornberger et al., 2009 | N | Y | Y | Y | Y | Y | Y | N |
| Jacus et al., 2018 | Y | Y | Y | N | N | Y | Y | N |
| Janssen et al., 2013 | Y | Y | Y | Y | N | N/A | Y | P |
| Johns et al., 2009 | N | Y | Y | Y | N | Y | Y | P |
| Johns et al., 2012 | N | Y | Y | Y | N | Y | Y | P |
| Larsen et al., 2015 | Y | Y | Y | P | Y | Y | Y | N |
| Lillo et al., 2011 | P | Y | Y | Y | N | Y | N | P |
| Lillo et al., 2020 | Y | Y | Y | Y | N | Y | Y | N |
| Lozachmeur et al., 2019 | Y | Y | Y | Y | N | Y | Y | N |
| Macoir et al., 2013 | N | Y | Y | Y | N | N | Y | N |
| Mariano et al., 2018 | U | Y | Y | Y | N | Y | Y | N |
| Martyr et al., 2019 | Y | Y | Y | Y | N | Y | Y | N |
| Matias-Guiu et al., 2019 | U | Y | Y | Y | N | Y | N | N |
|  |  |  |  |  |  |  |  |  |
|  | **Sampling** | | |  | **Blinding**** | **Verification** | | **Attrition***** |
|  | Random or consecutive recruitment | Recruitment source | Demographic variables provided | Participant confounds investigated* |  | Published diagnostic criteria | Tested controls |  |
| Nash 2007 | Y | Y | Y | Y | N | Y | Y | N |
| O'Callaghan et al., 2013 | Y | Y | Y | Y | N | Y | Y | N |
| Obeso et al., 2011 | Y | Y | P | P | N | Y | Y | P |
| Pettit et al., 2013 | U | Y | P | Y | N | Y | Y | Y |
| Primativo et al., 2017 | U | U | Y | Y | N | Y | U | N |
| Robinson et al., 2021 | U | Y | Y | Y | N | Y | Y | N |
| Siquier & Andres 2022 | Y | Y | Y | Y | Y | Y | Y | N |
| Southi et al., 2018 | Y | Y | Y | Y | N | Y | Y | Y |
| Staios et al., 2013 | Y | Y | P | Y | N | Y | Y | N |
| Tanguy et al., 2022 | Y | Y | Y | Y | N | Y | Y | N |
| Taylor et al., 2013 | Y | Y | Y | Y | N | Y | Y | N |
| Tjokrowijoto et al., 2023 | Y | Y | Y | Y | N | Y | Y | P |
| Tse et al., 2020 | N | Y | Y | Y | N | Y | Y | N |
| Van den Berg et al., 2009 | Y | Y | Y | N | N | N | Y | N |
| Vandenbossche et al., 2013 | U | U | Y | Y | N | N | Y | N |
| Vestberg et al., 2019 | Y | Y | P | N | N | Y | Y | Y |
| Wong et al., 2019 | N | Y | Y | Y | N | Y | Y | N |

*Note.* The contamination domain was not included in the risk-of-bias assessment, as it is explicitly addressed in the predefined inclusion criteria for the study.

*A study that thoroughly investigates confounded variables, such as the sample's age and education, is considered to have low risk-of-bias as it accounts for and address factors that could potentially influence the study outcomes.

**The blinding domain pertains to the extent of blinding during the administration and

scoring of the neuropsychological tests. It investigates whether the administering

neuropsychologist, the test scorer, or both were blinded to the relevant information.

However, this domain was omitted in the workup of the study risk-of-bias categories

as it is hard to employ in neurodegenerative samples due to the overt nature of the

symptoms.

***The attrition domain examines the variability in participant numbers throughout the study. If unexplained and present, it is considered a high risk of bias. Conversely, if a reasonable explanation is provided for the lower number of participants, it is categorized as moderate (or partial) risk of bias.

Supplementary Figure S2. Traffic Light Plot for Study Risk-of-bias (McGuiness & Higgins, 2021)


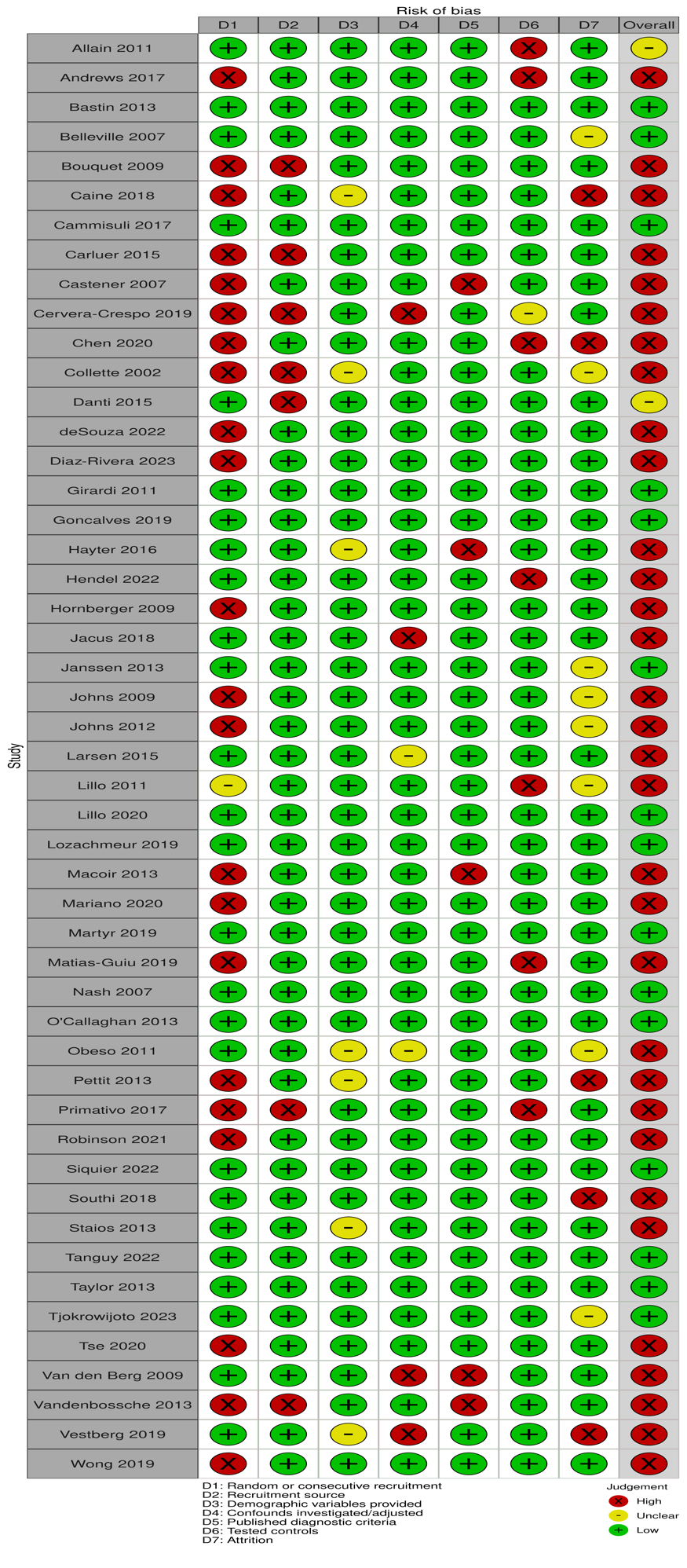


Supplementary Table S7. List of authors failed to respond

| Email addressed to | Study/studies |
| --- | --- |
| FRONTIER group (Hornberger, Irish and Piguet) | Chen, Y., Landin-Romero, R., Kumfor, F., Irish, M., Hodges, J. R., & Piguet, O. (2020). Cerebellar structural connectivity and contributions to cognition in frontotemporal dementias. *Cortex*, *129*, 57-67.    Hornberger, M., Geng, J., & Hodges, J. R. (2011). Convergent grey and white matter evidence of orbitofrontal cortex changes related to disinhibition in behavioral variant frontotemporal dementia. *Brain*, *134*(9), 2502-2512.    Irish, M., Hodges, J. R., & Piguet, O. (2013). Episodic future thinking is impaired in the behavioral variant of frontotemporal dementia. *Cortex*, *49*(9), 2377-2388.    Lillo, P., Savage, S., Mioshi, E., Kiernan, M. C., & Hodges, J. R. (2012). Amyotrophic lateral sclerosis and frontotemporal dementia: a behavioral and cognitive continuum. *Amyotrophic Lateral Sclerosis*, *13*(1), 102-109.    Leslie, F. V. C., Foxe, D., Daveson, N., Flannagan, E., Hodges, J. R., & Piguet, O. (2016). FRONTIER Executive Screen: a brief executive battery to differentiate frontotemporal dementia and Alzheimer's disease. *Journal of Neurology, Neurosurgery & Psychiatry*, *87*(8), 831-835.    Ramanan, S., Bertoux, M., Flanagan, E., Irish, M., Piguet, O., Hodges, J. R., & Hornberger, M. (2017). Longitudinal Executive Function and Episodic Memory Profiles in Behavioral-Variant Frontotemporal Dementia and Alzheimer’s Disease. *Journal of the International Neuropsychological Society*, *23*(1), 34-43.    Southi, N., Honan, C. A., Hodges, J. R., Piguet, O., & Kumfor, F. (2019). Reduced capacity for empathy in corticobasal syndrome and its impact on carer burden. *International Journal of Geriatric Psychiatry*, *34*(3), 497–503.    Strikwerda‐Brown, C., Mothakunnel, A., Hodges, J. R., Piguet, O., & Irish, M. (2019). External details revisited–A new taxonomy for coding ‘non‐episodic’content during autobiographical memory retrieval. *Journal of Neuropsychology*, *13*(3), 371-397.    Synn, A., Mothakunnel, A., Kumfor, F., Chen, Y., Piguet, O., Hodges, J. R., & Irish, M. (2018). Mental States in Moving Shapes: Distinct Cortical and Subcortical Contributions to Theory of Mind Impairments in Dementia. *Journal of Alzheimer's Disease*, *61*(2), 521-535. |
| van de Pavert | van de Pavert, S. H., Muhlert, N., Sethi, V., Wheeler-Kingshott, C. A., Ridgway, G. R., Geurts, J. J., Ron, M., Yousry, T. A., Thompson, A. J., Miller, D. H., Chard, D. T., & Ciccarelli, O. (2016). DIR-visible grey matter lesions and atrophy in multiple sclerosis: partners in crime?. *Journal of Neurology, Neurosurgery, and Psychiatry*, *87*(5), 461–467. |
| Caballero | Caballero, J. A., Auclair Ouellet, N., Phillips, N. A., & Pell, M. D. (2022). Social decision-making in Parkinson’s disease. *Journal of Clinical and Experimental Neuropsychology*, *44*(4), 302-315. |
| Ghosh | Ghosh, B. C., Carpenter, R. H., & Rowe, J. B. (2013). A longitudinal study of motor, oculomotor and cognitive function in progressive supranuclear palsy. *PloS One*, *8*(9), e74486. |
| Elamin | Phukan, J., Elamin, M., Bede, P., Jordan, N., Gallagher, L., Byrne, S., Lynch, C., Pender, N., & Hardiman, O. (2012). The syndrome of cognitive impairment in amyotrophic lateral sclerosis: a population-based study. *Journal of Neurology, Neurosurgery, and Psychiatry*, *83*(1), 102–108. |
| Cipolotti | Foley, J. A., Lancaster, C., Poznyak, E., Borejko, O., Niven, E., Foltynie, T., Abrahams, S., & Cipolotti, L. (2019). Impairment in Theory of Mind in Parkinson's Disease Is Explained by Deficits in Inhibition. *Parkinson's Disease*, *2019*, 5480913. |
| Williams | Migo, E. M., Mitterschiffthaler, M., O’Daly, O., Dawson, G. R., Dourish, C. T., Craig, K. J., Simmons, A., Wilcock, G. K., McCullosh E., Jackson, S. H. D., Kopelman, M. D., Williams, S. C. R., & Morris, R. G. (2015). Alterations in working memory networks in amnestic mild cognitive impairment. *Aging, Neuropsychology, and Cognition,* 22(1), 106-127. |
| Jacus | Jacus, J. P., Gély-Nargeot, M. C., & Bayard, S. (2018). Ecological relevance of the Iowa gambling task in patients with Alzheimer's disease and mild cognitive impairment. *Revue Neurologique*, *174*(5), 327-336. |
| Vallet (Hudon responded and deferred email to Vallet however no response afterwards) | Vallet, G. T., Hudon, C., Simard, M., & Versace, R. (2013). The disconnection syndrome in the Alzheimer's disease: the cross-modal priming example. *Cortex*, *49*(9), 2402–2415. |
| De Souza | de Souza, L. C., Bertoux, M., Radakovic, R., Hornberger, M., Mariano, L. I., Resende, E. D. P. F., Quesque, F., Guimarães, H. C., Gambogi, L. B., Tumas, V., Camargos, S. T., Cardoso, F. E. C., Teixeira, A. L., & Caramelli, P. (2022). I'm looking through you: Mentalizing in frontotemporal dementia and progressive supranuclear palsy. *Cortex*, *155*, 373-389.    Lillo, P., Caramelli, P., Musa, G., Parrao, T., Hughes, R., Aragon, A., Valenzuela, D., Cea, G., Aranguiz, R., Guimarães, H. C., Rousseff, L., Gambogi, L. B., Mariano, L. I., Teixeira, A. L., Slachevsky, A., & de Souza, L. C. (2020). Inside minds, beneath diseases: social cognition in amyotrophic lateral sclerosis-frontotemporal spectrum disorder. *Journal of Neurology, Neurosurgery, and Psychiatry*, *91*(12), 1279–1282.    Moura, M. V. B., Mariano, L. I., Teixeira, A. L., Caramelli, P., & de Souza, L. C. (2021). Social cognition tests can discriminate behavioral variant frontotemporal dementia from alzheimer’s disease independently of executive functioning. *Archives of Clinical Neuropsychology*, *36*(5), 831-837. |
| Rochester | Rochester, L., Hetherington, V., Jones, D., Nieuwboer, A., Willems, A. M., Kwakkel, G., & Van Wegen, E. (2005). The effect of external rhythmic cues (auditory and visual) on walking during a functional task in homes of people with Parkinson's disease. *Archives of Physical Medicine and Rehabilitation*, *86*(5), 999–1006. |
| Monchi | Yoon, E. J., Ismail, Z., Hanganu, A., Kibreab, M., Hammer, T., Cheetham, J., Kathol, I., Sarna, J. R., Martino, D., Furtado, S., & Monchi, O. (2019). Mild behavioral impairment is linked to worse cognition and brain atrophy in Parkinson disease. *Neurology*, *93*(8), e766-e777. |
| Debert | Joyce, J. M., Monchi, O., Ismail, Z., Kibreab, M., Cheetham, J., Kathol, I., Sarna, J, Martino, D., & Debert, C. T. (2020). The impact of traumatic brain injury on cognitive and neuropsychiatric symptoms of Parkinson’s disease. *International Review of Psychiatry*, *32*(1), 46-60. |
| McMahon | Isaacs, M. L., McMahon, K. L., Angwin, A. J., Crosson, B., & Copland, D. A. (2019). Functional correlates of strategy formation and verbal suppression in Parkinson's disease. *NeuroImage: Clinical*, *22*, 101683. |
| Foley | Foley, J. A., Niven, E. H., Abrahams, S., & Cipolotti, L. (2021). Phonemic fluency quantity and quality: Comparing patients with Progressive Supranuclear Palsy, Parkinson's disease and focal frontal and subcortical lesions. Neuropsychologia, *153*, 107772. |

Supplementary Figure S3. Publication bias analyses for Inhibition Errors score

AD vs. healthy controls


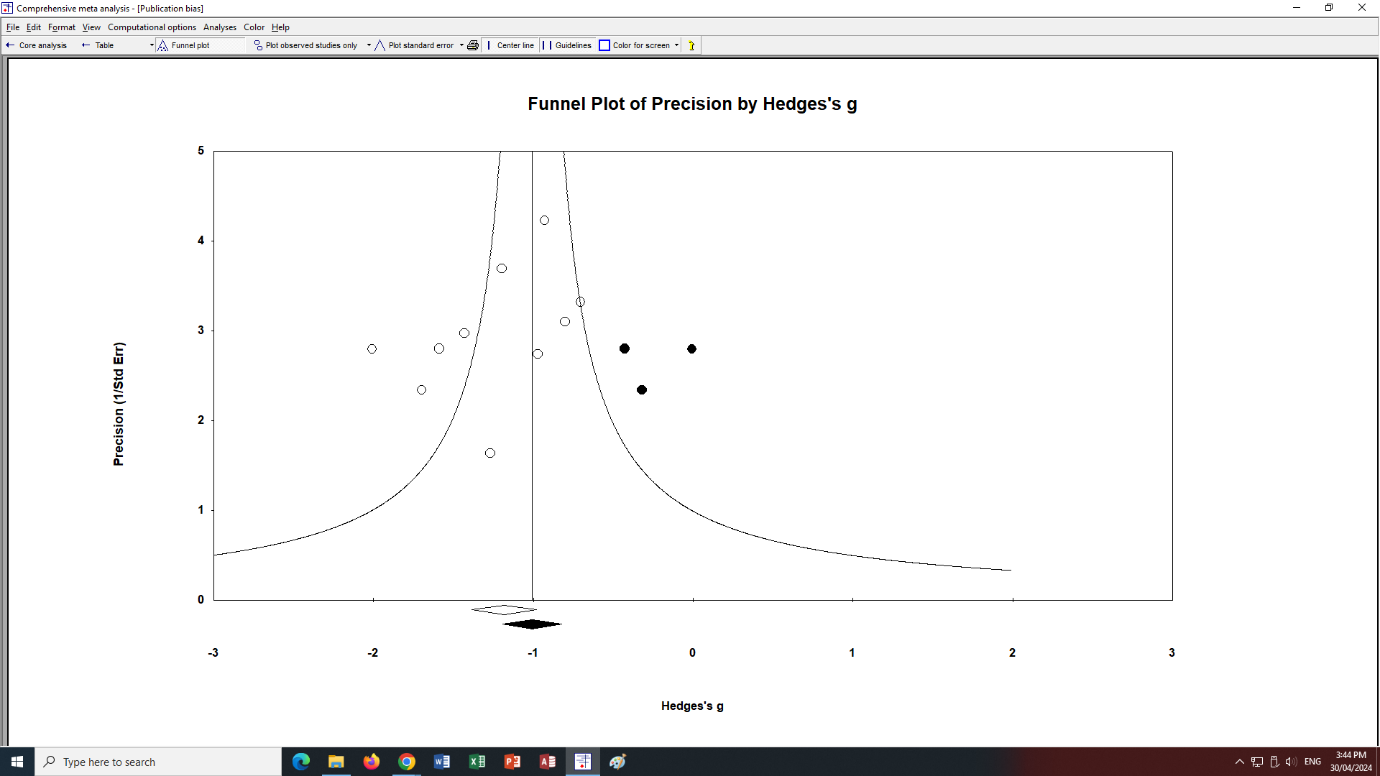


PD vs. healthy controls


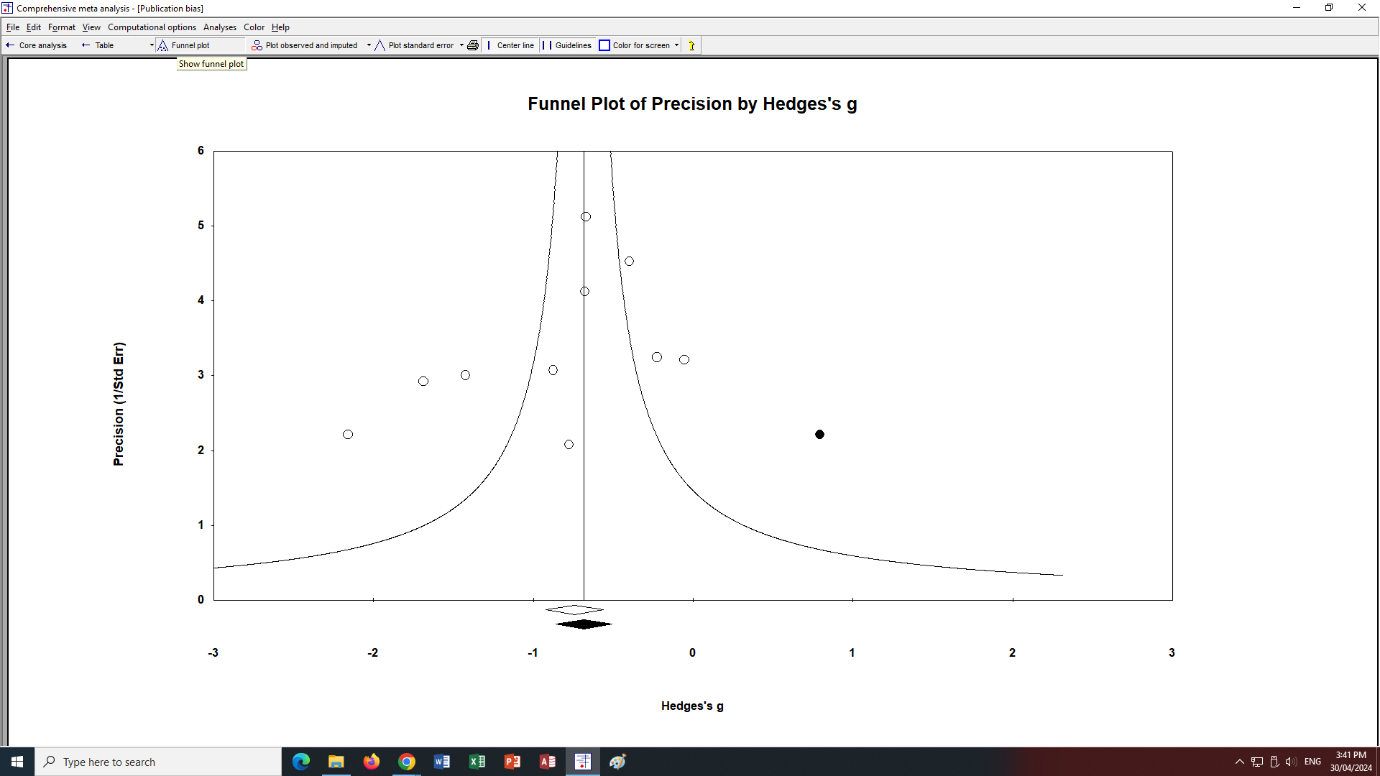

Supplement: Supplementary file 1 — Supplementary file1 (DOCX 1.51 MB) [file 11065_2025_9658_MOESM1_ESM.docx]
